# A comparison of full model specification and backward elimination of potential confounders when estimating marginal and conditional causal effects on binary outcomes from observational data

**DOI:** 10.1002/bimj.202100237

**Published:** 2022-05-12

**Authors:** Kim Luijken, Rolf H. H. Groenwold, Maarten van Smeden, Susanne Strohmaier, Georg Heinze

**Affiliations:** ^1^ Department of Clinical Epidemiology Leiden University Medical Center Leiden The Netherlands; ^2^ Department of Biomedical Data Sciences Leiden University Medical Center Leiden The Netherlands; ^3^ Julius Center for Health Sciences and Primary Care University Medical Center Utrecht University of Utrecht Utrecht The Netherlands; ^4^ Section for Clinical Biometrics Center for Medical Statistics Informatics and Intelligent Systems Medical University of Vienna Vienna Austria; ^5^ Department of Epidemiology Center for Public Health Medical University of Vienna Vienna Austria

**Keywords:** backward elimination, causal inference, confounder selection

## Abstract

A common view in epidemiology is that automated confounder selection methods, such as backward elimination, should be avoided as they can lead to biased effect estimates and underestimation of their variance. Nevertheless, backward elimination remains regularly applied. We investigated if and under which conditions causal effect estimation in observational studies can improve by using backward elimination on a prespecified set of potential confounders. An expression was derived that quantifies how variable omission relates to bias and variance of effect estimators. Additionally, 3960 scenarios were defined and investigated by simulations comparing bias and mean squared error (MSE) of the conditional log odds ratio, log(cOR), and the marginal log risk ratio, log(mRR), between full models including all prespecified covariates and backward elimination of these covariates. Applying backward elimination resulted in a mean bias of 0.03 for log(cOR) and 0.02 for log(mRR), compared to 0.56 and 0.52 for log(cOR) and log(mRR), respectively, for a model without any covariate adjustment, and no bias for the full model. In less than 3% of the scenarios considered, the MSE of the log(cOR) or log(mRR) was slightly lower (max 3%) when backward elimination was used compared to the full model. When an initial set of potential confounders can be specified based on background knowledge, there is minimal added value of backward elimination. We advise not to use it and otherwise to provide ample arguments supporting its use.

## 1 INTRODUCTION

Identification of causal effects from observational data relies on proper control for confounding. It is generally advised that confounders are determined based on the causal structure of the data, about which one may possess background knowledge, or one could at least make defendable assumptions (Ding & Miratrix, 2015; Greenland, 2003; Hernán & Robins, 2020; VanderWeele, 2019), and that automated covariate selection methods, such as stepwise selection and backward elimination, should be avoided as they can lead to seriously biased estimated effect sizes and underestimation of statistical uncertainty by model‐based confidence intervals (CIs) (Heinze et al., 2018; Leeb & Pötscher, 2005; Moosavi et al., 2021).

Despite these warnings, automated selection procedures for selection of confounders remain widely applied (Ali et al., 2015; Groenwold et al., 2008; Hemkens et al., 2018; Klein‐Geltink et al., 2007; Pouwels et al., 2016; Talbot & Massamba, 2019; Walter & Tiemeier, 2009). One reason for the popularity may be that, at least in theory, using automated selection as an add‐on to selection of potential confounders based on background knowledge may lead to improved efficiency (Greenland et al., 2016; VanderWeele, 2019; VanderWeele & Shpitser, 2011). For instance, backward elimination has occasionally been reported to improve estimation in terms of mean squared error (MSE) of the effect estimator (Dunkler et al., 2014). Limited guidance exists about when backward elimination could be beneficial in observational studies in which confounding adjustment is needed (Greenland, 2008; Vansteelandt et al., 2012; Witte & Didelez, 2019).

The aim of the current study was to extend recommendations for practicing statisticians on the use or avoidance of automated variable selection for descriptive models provided by Heinze et al. (2018) to a causal inference context. Specifically, we compare the efficiency of causal effect estimation by multivariable modeling in observational studies when fitting a model with all potential confounders (full model) compared to using backward elimination (see Box 1). Out of the many available methods for variable (or confounder) selection, we focus on backward elimination, because it is widely implemented in statistical software packages and is often considered superior to alternatives such as univariable screening, forward and stepwise selection (Sauerbrei et al., 2020). We focus on outcome‐oriented selection of confounders, meaning that exposure‐oriented selection procedures, for instance, as part of propensity score methods, are beyond the scope of this article. Furthermore, we assume that sufficient clinical expertise is available to specify an outcome model with covariates presumably related to the exposure and/or outcome free of mediators and colliders. This model is assumed to include at least all such covariates and to correctly specify all nonlinear covariate–outcome relations but may include covariates only related to the exposure (instruments) and/or true confounders, or irrelevant covariates.

In Section 2, we present comparative analyses of a motivating example. In Section 3, we discuss arguments in favor of and against the use of backward elimination as a means of automated selection among potential confounders. In Section 4, we perform simulation studies to investigate whether there is a benefit of using a backward‐elimination estimator compared to a full‐model estimator to estimate the target causal effect. We end with a discussion of the implications for clinical research.

**Box 1: Motivation to compare backward elimination of potential confounders neutrally with a full model**

- After identifying a set of potential confounders, uncertainty about the causal role of some covariates may remain. Backward elimination can reduce the adjustment set to arrive at a more precise estimate, possibly by introducing bias.
- The disjunctive cause criterion by VanderWeele and Shpitser can guide confounder selection (VanderWeele & Shpitser, 2011). This criterion states to control for covariates that are either a cause of the exposure or a cause of the outcome, which may lead to adjustment for instrumental variables. Therefore, they recommended implementing backward elimination or forward selection to eliminate such variables. On the other hand, Vansteelandt and colleagues argued that instrumental variables should not necessarily be eliminated from the adjustment set, because the uncertainty they introduce on the estimated exposure effect may reflect lack of information about the effect of interest (Vansteelandt et al., 2012).
- Greenland and colleagues proposed to compare a model adjusted for a sufficient set of confounders where one confounder is deleted by hand to a full model by estimating the change in MSE that was illustrated in an empirical data set (Greenland et al., 2016). As similar bias and variance considerations apply to backward elimination, it is worthwhile to compare a full model and use of backward elimination in more settings.
- Backward elimination has been reported to improve estimation in terms of MSE of the effect estimator (Dunkler et al., 2014).

## 2 MOTIVATING EXAMPLE: CORONARY ARTERY BYPASS GRAFTING STUDY

We illustrate confounder selection using a study that investigated the causal effect of a computer tomography angiography (CTA) examination of the main coronary artery prior to coronary artery bypass grafting (CABG) surgery on the postoperative stroke risk of a patient (Sandner et al., 2020). We used a simulated data set based on the empirical data (details in Sandner et al., 2020) that was previously used for methodological work (Gregorich, 2018). In the simulated data set, the sample size and relationships between the variables were preserved and similar to the original data set. In Supporting Information File 1, we provide R code to allow replication of this example.

### 2.1 Defining causal estimands

We defined two research questions and the corresponding estimands (Goetghebeur et al., 2020). The first research question compared the risk of postoperative stroke for patients with known characteristics when refraining from screening for aortic disease using CTA prior to CABG surgery versus the risk when patients were screened using CTA. The causal contrast, no CTA screening versus CTA screening given a set of characteristics, can, for instance, be expressed as a conditional risk difference, a conditional risk ratio, or a conditional odds ratio (cOR). We defined the estimand as the cOR.

The second question of interest concerned the effect of not exposing an entire target population to CTA screening versus exposing everyone to CTA screening on postoperative stroke risk. The causal contrast could, for instance, be expressed as a marginal risk difference, a marginal risk ratio (mRR), or a marginal odds ratio. We defined the estimand as the mRR.

### 2.2 Linking the observed data to the estimand

To evaluate whether the causal effect of CTA screening on postoperative stroke risk can be identified from observational data, we specified the research problem in terms of potential outcomes (Hernán & Robins, 2020). Let Y^CTA^ denote the potential outcome that would have been observed for an individual if they were set to endure CTA screening, possibly counter to fact. We assume that the set of measured characteristics is sufficient to invoke the assumption that the potential outcomes Y^CTA^ are independent of CTA screening status given the set of measured characteristics, that is, conditional exchangeability is assumed. The exposure of CTA screening is considered sufficiently well defined to invoke the consistency assumption.

Establishing a set of covariates to invoke conditional exchangeability in a clinical scenario remains challenging, in particular, because knowledge about the causal mechanism under study is often incomplete. Heinze and colleagues recommended to generate an initial working set of covariates based on clinical expertise and background knowledge, without yet using the data set at hand (Harrell Jr, 2015; Heinze et al., 2018). In studies of causal inference, it is often helpful to visualize assumed causal dependencies between covariates, where the level of formalization of those dependencies may sometimes reach that of a directed acyclic graph (DAG) (we refer to Tennant et al., 2021 for recommendations on implementation). In doing so, a researcher explicates knowledge about variables that are *irrelevant* to the study question, as leaving out variables is a stronger assumption than including them. Accordingly, for covariates that are included in an initial working set, many decisions are still to be made regarding their causal role and relevance.

In the original study (Sandner et al., 2020), the initial working set contained 23 measured covariates that described the health state of a patient just before the decision to perform CTA or not. Detailed causal assumptions that could be represented in a DAG were not supported by the cross‐sectional assessment of these covariates, but we could exclude collider stratification bias or presence of mediators based on background knowledge when using these covariates as a confounding adjustment set.

### 2.3 Estimation of causal effects and confidence intervals

We estimated the cOR by the exponentiated regression coefficient of no CTA screening in a multivariable logistic regression model with Firth's correction (Firth, 1993; Heinze & Schemper, 2002; Puhr et al., 2017) (CIs based on profile penalized likelihood) including the 23 covariates specified in the initial working set. Notably, when backward elimination is used for estimation of causal effects, it can be considered an estimation tool to estimate the specified estimand with improved precision, yet probably introducing bias. The conditional exchangeability assumption is invoked conditional on all covariates specified in the full model, yet backward elimination sets some of the covariate–outcome associations to zero. The resulting cOR is interpreted conditional on all covariates specified in the full model.

We estimated the mRR based on predictions of potential outcomes from that multivariable logistic regression model (Austin, 2010; Greenland, 2004; Localio et al., 2007) (CIs based on 500 bootstrap samples using the percentile method). Additionally, we applied data‐driven selection of the 23 prespecified covariates by means of backward elimination at a significance level of 0.157 approximating selection by the Akaike information criterion (Heinze et al., 2018). For the backward‐elimination estimator, we contrasted “selected‐model” CIs, which condition on the finally selected covariates, to “global” bootstrap CI, where the selection process was repeated in each bootstrap resample. The selected‐model CIs were based on profile penalized likelihood for the cOR and computed from fitting the finally selected model in 500 bootstrap samples using the percentile method for the mRR.

In this example, backward elimination reduced the adjustment set by eight potential confounders. While for both cOR and mRR, the full‐model CIs were wider than the (invalid) selected model CIs, the global bootstrap CIs were the widest (Table 1). Clearly, additional variability arises from the uncertainty in the selection that must be captured by repeating the selection process in each bootstrap resample. Heinze and colleagues proposed to evaluate bias and added uncertainty by two bootstrap‐based measures, relative conditional bias (RCB), and root mean squared difference ratio (RMSDR) (Heinze et al., 2018; Wallisch et al., 2021). In the current example, the RCB for the log cOR was −1.3% and RMSDR was 1.06. The RCB for the log mRR was −2.6% and RMSDR was 1.07. These measures also indicated a possible variance inflation by using backward elimination.

**TABLE 1:** Results for the CABG study

Estimand	Model	Estimate	Confidence interval estimation approach	95% Confidence interval	Confidence interval width (upper/lower)
cOR	Full	4.48	PPL	[2.10, 10.01]	4.77
	Selected	3.83	Invalid: Selected‐model PPL	[1.99, 7.77]	3.90
			Global bootstrap	[2.10, 11.25]	5.36
mRR	Full	3.68	Bootstrap	[2.02, 7.09]	3.51
	Selected	3.24	Invalid: Selected‐model bootstrap	[1.87, 6.19]	3.31
			Global bootstrap	[1.93, 7.17]	3.72

cOR, conditional odds ratio; mRR, marginal risk ratio; PPL, profile penalized likelihood.

## 3 USE OF AUTOMATED COVARIATE SELECTION

### 3.1 Arguments in favor of automated selection of confounders

Bias and variance of an effect estimator can be combined in a single measure; the MSE. The MSE can be interpreted as the expected value of the squared distance of an estimate to the true value, which can be alternatively expressed as *MSE = bias^2^ + variance*. For a linear regression model, the value of omitting a covariate in terms of reducing the MSE of an effect estimator can be quantified directly (see Supporting Information File 2). We provide a simplified representation of this principle here that extends to settings with binary outcomes.

Consider a setting with an outcome, an exposure and one covariate. The effect of the exposure on the outcome is evaluated under two estimation strategies: “always include the covariate” (full) versus “always omit the covariate” (omit). Assuming that the bias in the exposure effect estimator of the “full” strategy is 0, in terms of MSE, we find a benefit in omitting the covariate if, for the effect of the exposure on the outcome, the following inequality holds:

(1) $$Bias_{omit}^{2}<\;Variance_{full}-Variance_{omit}.$$

**FIGURE 1:**
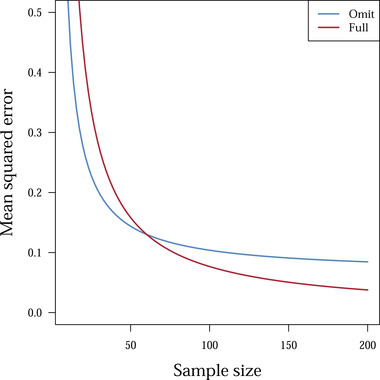
Illustration of the bias‐variance trade‐off for the ordinary least squares estimator of the exposure effect when including (Full) or omitting (Omit) covariate *L*. The blue and red line are computed using expressions for the mean squared error under the “Omit” and “Full” strategy, respectively (Supporting Information File 2). The value of *n* for which the reduced variance by omitting *L* outweighs the increase in squared bias is around 60. This illustrates inequality (1) in the main text for a linear model and ordinary least squares estimation. For sample sizes *< *60, omission of the covariate resulted in a lower mean squared error of the exposure effect estimator in a linear setting

If (1) holds, the reduced variance of the “omit” strategy outweighs the increase in squared bias, and thus, there is a benefit of omitting the covariate in terms of MSE, and hence produces a more efficient estimate. If we ignore a possible small sample bias (Cordeiro & McCullagh, 1991; Schaefer, 1983), only the right‐hand side of (1) is inversely proportional to sample size. Thus, there should be a threshold sample size *n*, such that (1) holds for all values smaller than that *n*. Figure 1 illustrates this phenomenon. Figure S2‐2 and S2‐3 (in Supporting Information File 2) illustrate that *n* increases with a stronger association between the exposure and the covariate, with a weaker association between the outcome and covariate, and with a lower variance of the exposure variable.

In a causal framework, the covariate might be considered to invoke conditional exchangeability. However, when the reduction in variance by omitting the covariate outweighs the increase in squared bias, covariate omission may be thought of as a way to estimate the same estimand using a possibly more precise estimator.

### 3.2 Arguments against automated selection of confounders

Selection of variables by statistical procedures is sometimes incorrectly thought to be a prerequisite for model building (Heinze & Dunkler, 2017). However, a “statistically significant” result neither confirms whether a covariate is indeed a confounder, nor does insignificance prove that it is not. A well‐known counterargument against use of data‐driven selection of confounders is that the causal structure of the data cannot be derived from observed associations only. For example, a covariate has a different causal status being a *confounder* compared to being a *mediator*, but in both cases, it may be statistically associated with the exposure and/or outcome. Automated covariate selection procedures based on statistical associations only could result in inappropriate adjustment, selection bias, or reduction of precision of the exposure effect estimate (Heinze & Dunkler, 2017; Sun et al., 1996). It has been claimed that postselection inference cannot be valid at all (Leeb & Pötscher, 2005). As research on this issue is ongoing (Belloni et al., 2016; Berk et al., 2013), neutral comparison studies and user‐friendly implementations are still lacking (Kammer et al., 2020), and hence, its advances are hardly accessible to epidemiologists. Frequentist statistical theory assumes that the parameters to be estimated in a model are fixed before observing the data, while variable selection involves the data in the selection process, meaning that the model is not fixed a priori. Consequently, CIs based on the selected model are no longer valid and often underestimate uncertainty in the effect estimator (Berk et al., 2013; Heinze et al., 2018; Sauerbrei et al., 2020).

Finally, there is no one‐size‐fits‐all implementation of automated covariate selection (Heinze et al., 2018; Sauerbrei et al., 2020) and recommendations on covariate selection may not be applicable to a particular study. Choices regarding covariate selection should strongly depend on the aim of a study, which could be causal inference, prediction, or description (Hernán et al., 2019; Shmueli, 2010). Statistical texts that explain variable selection do not always relate implementation of the procedure to those distinct research aims (Shmueli, 2010).

## 4 SIMULATIONS

### 4.1 Simulation design

**Aim**: We examined the effect of backward elimination versus full model specification on the efficiency of causal effect evaluation in simulation studies. First, we performed a proof‐of‐concept simulation (Experiment 1) to confirm inequality (1). Additionally, we studied the value of backward elimination in efficiency of causal effect estimation in more complex and realistic settings (Experiment 2). Application of backward elimination was considered an estimation tool to estimate the specified estimands with improved precision, yet probably introducing bias.

**Data‐generating mechanisms**: The generated data consisted of a binary outcome, *Y*, a binary exposure, *A*, and a set of continuous covariates, *L*. The set of covariates was free of mediators and colliders and was the starting point for all backward elimination procedures. In Experiment 1, the generated data contained a single continuous covariate next to the exposure and outcome. The exposure effect was null, the sample size was set to 60 or 120 and the event fraction (i.e., Pr(*Y *= 1)) was set to 0.5 or 0.2. The conditional associations *A*−*L* and *Y* −*L* varied between 0 and 0.5 on a log‐odds scale. A total of 144 scenarios were evaluated. In Experiment 2, the log(cOR) of the exposure was either log(1) or log(1.5). *L* consisted of 24 continuous covariates from a multivariate normal distribution with mean 0 and a variance–covariance matrix with 1s on the diagonal and 0.3 on all off‐diagonal elements. The set consisted of a mix of 12–24 true confounders, 0–12 (near) instrumental variables, 0–12 (near) predictors of the outcome, and 0–12 noise variables, where the number of each covariate type was varied across simulation scenarios (see Table 2). The expected number of events was set to 50 or 200 and the expected event fraction was set to 0.2 or 0.03, resulting in samples with 250, 1667, 1000, or 6667 observations. Table 2 presents the values of other simulation parameters. A total of 3960 scenarios were evaluated.

**TABLE 2:** Simulation parameters of experiment 2

Parameter	Value
Conditional exposure‐outcome effect	0, log(1.5)
Fixed confounders: conditional log odds ratio confounder‐exposure association^a^	log(1.05)
Fixed confounders: conditional log odds ratio confounder‐outcome association^a^	log(1.05)
Mixture of covariates: conditional log odds ratio covariate–exposure association (four sets of three covariates)^a^	0, log(1.05), log(1.2)
Mixture of covariates: conditional log odds ratio covariate–outcome association (four sets of three covariates)^a^	0, log(1.05), log(1.2)
Covariate correlation across all 24 covariates	0.3
Number of events	50, 200
Expected event fraction	0.2, 0.03

^a^ Of the 24 continuous covariates, 12 were assumed to be fixed confounders, and 12 represented a mixture of true confounders (log(1.2)), (near‐) instrumental variables (log(1.05)), (near‐)predictors of the outcome (log(1.05)), and noise variables (0). In each data set, the number of respective covariate types was determined by the combination of conditional coavariate exposure/outcome parameters.

**Target estimand**: The estimands were the cOR and the mRR of the causal effect of *A* on *Y*.

**Methods**: The cOR was obtained from logistic regression models estimated using Firth's Logistic regression with intercept correction (FLIC) to avoid introduction of finite sample bias (Firth, 1993; Heinze & Schemper, 2002; Puhr et al., 2017) and issues with separation in the simulation (van Smeden et al., 2016). The mRR was estimated using FLIC models that estimated potential outcomes (Austin, 2010; Greenland, 2004; Localio et al., 2007). Estimates were evaluated on a logarithmic scale because of the asymmetrical nature of ORs and RRs. Simulations were performed using R statistical software version 3.6.2.(R Core Team, 2013) using the package logistf (Heinze et al., 2020) to implement Firth's correction. In Experiment 1, the MSE of the log($\hat{cOR}$) and log($\hat{mRR}$) was evaluated under two estimation strategies: “always include covariate L” versus “always omit covariate L.” In Experiment 2, we evaluated the MSE of the log($\hat{cOR}$) and log($\hat{mRR}$) obtained using a full model versus using backward elimination with cut‐off value *p* = 0.157 (corresponding with using the Akaike information criterion) (Harrell Jr, 2015). We obtained the true mRR for each scenario by a large sample approximation (*N *= 1,000,000).

**Performance measures**: The MSE was defined as the average squared difference between the estimated log($\hat{cOR}$) and true log(cOR) or the estimated log($\hat{mRR}$) and true log(mRR) averaged per scenario over the simulation runs (10,000 for Experiment 1; 1000 for Experiment 2). We compared the full and selected model in terms of relative efficiency of the log($\hat{cOR}$) and log($\hat{mRR}$), which was computed as a ratio of the MSE obtained from the backward elimination procedure divided by the MSE obtained from the full model.

This simulation design was reported following previous recommendations (Morris et al., 2019). All R code for simulations is available at https://github.com/Kluijken/CI_CovSel.

### 4.2 Results

**FIGURE 2:**
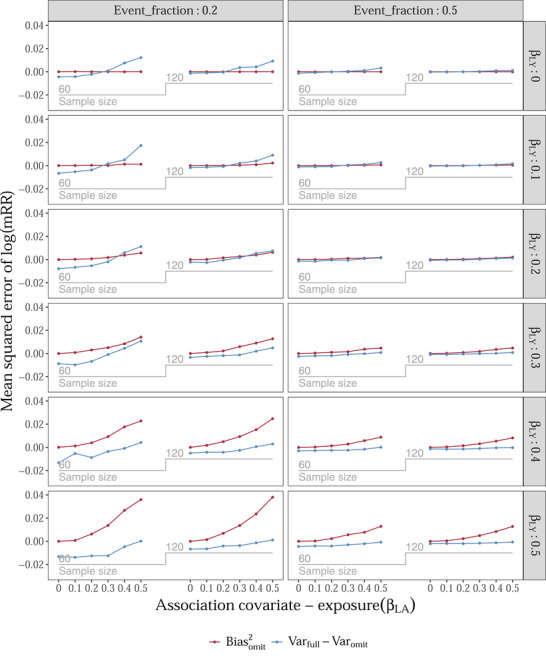
Results of simulation Experiment 1 for the marginal risk ratio (mRR). A single covariate *L* acts as a confounder, (near‐)instrumental variable, (near‐)predictor of the outcome, or noise variable in a setting where a binary exposure has a true null effect on a binary outcome. The squared bias and difference in variance is compared when *L* is always included or always omitted, illustrating principle (1) in the main text. $\beta_{LA}$ and $\beta_{LY}$ refer to the conditional log odds ratio of the covariate–exposure and covariate–outcome association, respectively. This figure was created using the looplot package (Kammer, 2020)

**Experiment 1**: Inequality (1) held for most (90%) of the simulated scenarios in Experiment 1 for the log($\hat{cOR}$) and for 20% of the scenarios regarding the log($\hat{mRR}$). Hence, regarding the cOR, omitting the covariate was often more beneficial in terms of MSE than including it (see Supporting Information File 3). Regarding the mRR, including the covariate was often more beneficial in terms of MSE than omitting it (Figure 2). Omitting the covariate was beneficial in terms of MSE only when the covariate was an instrument or a near‐instrument. The benefit of omitting was larger when the event fraction was lower, 0.2 instead of 0.5, and, as expected, when sample size was lower, 60 compared to 120.

**Experiment 2**: In Experiment 2, the median relative efficiency of the log($\hat{cOR}$) across all scenarios was 1.04, indicating that the MSE was on average lower for the full model than the selected model. The median relative efficiency of the log($\hat{mRR}$) across all scenarios was 1.05. Across all 3960 scenarios, the bias of the full model was zero for both the log($\hat{cOR}$) and log($\hat{mRR}$), whereas the average bias across all scenarios of the backward eliminated model was 0.03 for the log($\hat{cOR}$) and 0.02 for the log($\hat{mRR}$), compared to 0.56 and 0.52 for log($\hat{cOR}$) and log($\hat{mRR}$), respectively, for a model without any covariate adjustment. We found 112 scenarios (2.8%) for cOR and 47 scenarios (1.1%) for mRR in which the MSE was lower for the selected than the full models.

Closer examination of the 112 scenarios in which the $\hat{cOR}$ estimated using backward elimination showed lower MSE than the full model revealed that 100 scenarios included at least three full instrumental variables and 37 scenarios included at least three noise variables (see Table 3). In these scenarios, the increased efficiency remained small, with a minimal relative efficiency of 0.97, meaning that the MSE when backward elimination was applied was only 3% lower than the MSE of the full model in the most beneficial setting. In the 47 scenarios in which the log($\hat{mRR}$) estimated using backward elimination showed lower MSE than the full model, we found that 42 scenarios included at least three full instrumental variables and 18 scenarios included at least three noise variables (see Table 4). Again, the increased efficiency remained small, with a minimal relative efficiency of 0.97. Full results of the simulations are presented in Supporting Information File 4. On request of one of the reviewers, we also added results on the marginal odds ratio and marginal risk difference, on coverage and on the number of times a true confounder was eliminated.

**TABLE 3:** Summary of simulation Experiment 2; results for the conditional odds ratio (cOR). Each row represents 495 scenarios with varying associations between the covariates and the exposure and/or outcome. Mean bias indicates the average bias of the log(cOR) for the full and backward eliminated model, respectively. Relative efficiency of the mean squared error (MSE) of the cOR is computed as a ratio of the backward elimination MSE divided by the full model MSE

Conditional exposure effect	Event fraction	Number of events	Mean bias full	Mean bias BE	Median relative efficiency	Minimum relative efficiency	Maximum relative efficiency	Number of scenarios MSE (cOR) BE < full	At least 3 IVs in DGM	At least 3 noise variables in DGM	No IVs or noise in DGM
0	0.20	50	0.00	0.04	1.08	1.00	1.20	0	0	0	0
0	0.20	200	0.00	0.02	1.03	0.98	1.13	10	9	3	0
0	0.03	50	0.00	0.03	1.03	0.98	1.10	6	6	1	0
0	0.03	200	0.00	0.02	1.02	0.97	1.08	42	40	14	0
log(1.5)	0.20	50	0.00	0.06	1.09	1.02	1.27	0	0	0	0
log(1.5)	0.20	200	0.00	0.03	1.04	0.98	1.13	8	8	3	0
log(1.5)	0.03	50	0.00	0.04	1.04	0.99	1.10	7	5	3	1
log(1.5)	0.03	200	0.00	0.02	1.02	0.98	1.09	39	32	13	3
**Overall results**			0.00	0.03	1.04	0.97	1.27	112	100	37	4

BE, backward elimination; cOR, conditional odds ratio; DGM, data‐generating mechanism; IV, instrumental variable; MSE, mean squared error

**TABLE 4:** Summary of simulation Experiment 2; results for the marginal risk ratio (mRR). Each row represents 495 scenarios with varying associations between the covariates and the exposure and/or outcome. Mean bias indicates the average bias of the log(mRR) for the full and backward eliminated model, respectively. Relative efficiency of the mean squared error (MSE) of the mRR is computed as a ratio of the backward elimination MSE divided by the full model MSE

Conditional exposure effect	Event fraction	Number of events	Mean bias full	Mean bias BE	Median relative efficiency	Minimum relative efficiency	Maximum relative efficiency	Number of scenarios MSE (cOR) BE < full	At least three IVs in DGM	At least three noise variables in DGM	No IVs or noise in DGM
0	0.20	50	0.00	0.03	1.12	1.06	1.25	0	0	0	0
0	0.20	200	0.00	0.02	1.05	1.00	1.17	0	0	0	0
0	0.03	50	0.00	0.03	1.05	1.00	1.12	0	0	0	0
0	0.03	200	0.00	0.02	1.02	0.97	1.09	21	19	8	0
log(1.5)	0.20	50	‐0.02	0.03	1.12	1.05	1.31	0	0	0	0
log(1.5)	0.20	200	0.00	0.02	1.05	1.00	1.15	1	1	0	0
log(1.5)	0.03	50	‐0.01	0.03	1.05	1.01	1.12	0	0	0	0
log(1.5)	0.03	200	0.00	0.02	1.02	0.98	1.10	25	22	10	0
**Overall results**			0.00	0.02	1.05	0.97	1.31	47	42	18	0

BE, backward elimination; DGM, data‐generating mechanism; IV, instrumental variable; mRR, marginal risk ratio; MSE, mean squared error.

## 5 DISCUSSION

Our simulation results show that, compared to estimating a model with all prespecified confounders, application of backward elimination was unlikely to reduce the MSE of the exposure effect estimator (defined as the cOR and the mRR), while introducing a bias. We identified some settings in which the MSE of the effect estimators was lower with backward elimination than without, yet the reduction in MSE was small. The results are driven by two antagonist effects: an MSE‐reducing effect of omitting weak confounders, and an MSE‐increasing effect caused by additional uncertainties incurred by applying automated selection as explained by Heinze et al (2018).

Despite the vast literature on confounder selection, confusion around the topic of covariate selection in studies of causal effects remains. Our work adds to understanding the (lack of the) value of using backward elimination when estimating a causal effect using a moderate number of covariates. The derivation of the relation between bias and variance provided an analytical basis, while the simulations illustrated the implications for realistic finite‐sample scenarios. What is more, revitalizing the bias‐variance trade‐off discussion adds to existing applied causal research, which seems to be mainly focused on minimizing bias. Our findings support and extend previous recommendations on automated covariate selection. VanderWeele and Shpitser proposed to use the disjunctive cause criterion for confounder selection (VanderWeele & Shpitser, 2011). This criterion states to control for covariates that are either a cause of the exposure or a cause of the outcome, which may lead to adjustment for instrumental variables. Therefore, they recommended implementing backward elimination or forward selection to eliminate such variables. Our findings provide weak support for the use of variable selection in this case. In an overview and classification of covariate selection strategies, Witte and Didelez found that backward elimination performed well in terms of bias in the effect estimator in settings that contained strong confounders and instrumental variables and did not perform well when applied to a sufficient adjustment set in which each confounder was responsible for a small degree of confounding (Witte & Didelez, 2019). We found similar patterns in terms of the MSE of the effect estimator, irrespective of whether conditional or marginal effects are of interest. On the other hand, Vansteelandt and colleagues recommended against the use of automated covariate selection even when there is a potential efficiency gain by excluding an instrumental variable, because this would prevent overstating the precision with which a causal effect is known (Vansteelandt et al., 2012). Summarizing, the true number of irrelevant covariates and instruments included in the prespecified set of adjustment variables, and the strength of association of true confounders with the outcome greatly affect the relative performance of applying backward elimination. In practice, these conditions are usually unknown, but the more domain expertise is available to define the set, the less a researcher has to rely on data‐driven selection.

Our motivating example was typical for clinical observational studies where a set of covariates is available that accurately describes the health state of a subject just before the decision to perform an intervention or not, but where dependencies among these covariates are difficult to assess. Therefore, we only assumed that the set of covariates was free of mediators and that there was no unmeasured confounding. These assumptions were based on clinical expertise and allowed specification of an initial working set without explicitly specifying a full DAG. Under these conditions, backward elimination was applied to potentially increase the efficiency of the effect estimate by setting weak covariate effects to zero, but not to change the underlying assumptions. One complication is that the obtained conditional effect should be interpreted as conditional on the full set of potential confounders.

A limitation of our study is that we did not consider scenarios in which clinical expertise is not available. In many clinical settings, it is questionable whether the assumption of no residual confounding really holds. Furthermore, it is difficult to judge to what extent preselection can be reliably done. This depends on the novelty of a research field, and often, one will rely on previous research to derive assumptions. Doing so, researchers should be aware of inappropriate methodology, such as questionable conclusions stemming from observed bivariate associations, which typically do not reflect multivariable relations represented in a causal network (Sun et al., 1996). It is up to the researcher to explain to what extent preprocessing based on background knowledge is possible and hence whether data‐driven selection could be of added value.

Additionally, as our paper was intended to evaluate a common practice, we did not consider more sophisticated approaches for data‐driven confounder selection. Although backward elimination is an outcome‐oriented selection procedure, other approaches, such as Lasso‐penalized regression approaches (Ertefaie et al., 2018; Wilson & Reich, 2014), take into account both covariate–outcome and covariate–exposure relations. Such approaches might lead to more robust and efficient effect estimation compared to backward elimination; however, they are hardly ever used in epidemiological studies. We also excluded augmented backward elimination (Dunkler et al., 2014) and other novel approaches as we were either involved in developing these methods or lack the necessary expertise to apply them routinely. Finally, because the number of scenarios in our simulations was large, nuances in interpretation might be lost by averaging over subsets of the scenarios. Specific scenarios that are of particular interest to readers can be evaluated in detail using the simulation code that is publicly available through GitHub.

We conclude that backward elimination for confounder selection is unlikely to have added value when an initial set of covariates related to the exposure and/or outcome can be specified based on background knowledge. If researchers choose to perform backward elimination of potential confounders, selection should be justified, for example, because a large number of potential confounders are anticipated to function as (near‐)instruments, and the approach should be prespecified in a statistical analysis plan. Covariate selection based solely on statistical criteria should be avoided due to the possible selection of mediators and colliders. Irrespective of whether or not covariate selection strategies are being applied, we recommend to always provide information about the assumed causal structure, ideally by a depiction of assumed causal dependencies, but at least by excluding mediators and the possibility of unmeasured confounding.

## CONFLICT OF INTEREST

The authors have declared no conflict of interest.

### OPEN RESEARCH BADGES

This article has earned an Open Data badge for making publicly available the digitally‐shareable data necessary to reproduce the reported results. The data is available in the Supporting Information section.

This article has earned an open data badge “**Reproducible Research**” for making publicly available the code necessary to reproduce the reported results. The results reported in this article were reproduced partially due to their computational complexity.

## Supporting information



Supporting information

Supporting information

Supporting information

Supporting information

Supporting information

## Data Availability

All R code to generate simulation data and perform the analyses conducted in this manuscript is available at https://github.com/KLuijken/CI_CovSel and from the Supporting Information files. To facilitate replication, a detailed description of simulation output is provided in Supporting Information file 4.
