# Supplementary material for: A comparison of full model specification and backward elimination of potential confounders when estimating marginal and conditional causal effects on binary outcomes from observational data
Source: Biom J. 2022 May 12;66(1):2100237. doi: 10.1002/bimj.202100237 (PMC10952199; doi:10.1002/bimj.202100237)
Supplement: Supplementary file 1 — Supporting information [file BIMJ-66-0-s004.pdf]

```
#------------------------------------------------------------------------------#
# A comparison of full model specification and backward elimination of potential
#   confounders when estimating marginal and conditional causal effects on
#   binary outcomes from observational data
# Authors: K Luijken, R H H Groenwold, M van Smeden, S Strohmaier, G Heinze
# Author script: K Luijken
#
# Perform CABG example analysis
#------------------------------------------------------------------------------#

# This script describes the analysis on simulated data of the example CABG study
# presented in manuscript section 2. The following analyses are presented:
## conditional odds ratio of a full model, estimated using FLIC
## marginal risk ratio of a full model, estimated using predicted potential
#     outcomes of a FLIC model
## conditional odds ratio of a selected model, estimated using FLIC and backward
#     elimination
## marginal risk ratio of a selected model, estimated using predicted potential
#     outcomes of a FLIC model and backward elimination
## confidence intervals are obtained using bootstrap, in which the model select-
#     ion process is repeated
## computation of the measures of relative conditional bias and root mean
#     squared difference ratio.

# All analyses accompanying the manuscript can be found on https://github.com/
KLuijken/CI_CovSel

# Load librairies ----
#------------------------------------------------------------------------------#
library(logistf)
library(ggplot2)

# Generate simulated data ----
#------------------------------------------------------------------------------#
# (Optional: use simulated data, available from Github repository)

source(file = "./rcode/add-ons/simulated_CABG_example/simulate_data_Gregorich.R")
set.seed(20200618)
CABG_data<- generate_data(N = 2266,
              betaTr.zero = FALSE,
              avsu = FALSE)

# Conditional OR, FLIC, full model ----
#------------------------------------------------------------------------------#

# Estimate model
Firth_full   <- logistf(Postoperative.stroke ~ CT + Age + Gender + Smoker +
                         Diabetes.Control + CreaCl+ Dialysis + Hypertension +
                         Peripheral.Vascular.Disease + Cerebrovascular.Accident+
                         Cerebrovascular.Disease + Myocardial.Infarction +
                         Congestive.Heart.Failure + Angina.Type + Afib.flutter +
                         Number.of.Diseased.Coronary.Vessels +
                         Left.Main.Disease + Ejection.Fraction + Status +
                         Dyslipidemia + Lipid.Lowering + Previous.Valve +
                         Previous.Coronary.Artery.Bypass + Year.CABG,
                       data = CABG_data,
                       control = logistf.control(maxit = 200, maxstep = 5),
                       firth = TRUE, # set firth = FALSE for max.likelihood est.
                       pl = TRUE,    # compute profile likelihood CIs
                       flic = TRUE)  # Perform intercept correction
```

```
# Obtain coefficients + profile penalized likelihood CIs
cOR_full_Firth_Est <- round(exp(coef( Firth_full)["CT"]), digits = 2)
cOR_full_Firth_Low <- round(exp(confint(Firth_full)[2,1]), digits=2)
cOR_full_Firth_Up  <- round(exp(confint(Firth_full)[2,2]), digits=2)
paste0(cOR_full_Firth_Est,
       "(95% CI, ", cOR_full_Firth_Low,
       "; ", cOR_full_Firth_Up,")")

# Marginal RR, FLIC, full model ----
#------------------------------------------------------------------------------#

# Create potential outcome datasets
All_unexposed <- CABG_data
All_unexposed$CT <- 0
All_exposed <- CABG_data
All_exposed$CT <- 1

# Obtain predicted potential outcomes
PredA0 <- as.vector(predict(Firth_full,
                            newdata = All_unexposed, type = "response"))
PredA1 <- as.vector(predict(Firth_full,
                            newdata = All_exposed, type = "response"))

# Estimate Marginal RR
mRR_full_Firth_Est <- mean(PredA1)/mean(PredA0)

### NB: CIs are bootstrapped below, such that they can be combined with
### bootstrap estimation of CIs of the selected model

# Conditional OR, FLIC, backward elimination ----
#------------------------------------------------------------------------------#

# Estimate model
Firth_selected  <- backward(Firth_full,
                            scope = c("Age", "Gender", "Smoker",
                                      "Diabetes.Control", "CreaCl", "Dialysis",
                                      "Hypertension",
                                      "Peripheral.Vascular.Disease",
                                      "Cerebrovascular.Accident",
                                      "Cerebrovascular.Disease",
                                      "Myocardial.Infarction",
                                      "Congestive.Heart.Failure", "Angina.Type",
                                      "Afib.flutter",
                                      "Number.of.Diseased.Coronary.Vessels",
                                      "Left.Main.Disease", "Ejection.Fraction",
                                      "Status", "Dyslipidemia","Lipid.Lowering",
                                      "Previous.Valve",
                                      "Previous.Coronary.Artery.Bypass",
                                      "Year.CABG"),
                            slstay = 0.157,
                            trace = TRUE,
                            control = logistf.control(maxit = 200, maxstep = 5),
                            pl = TRUE)
Firth_selected      <- flic(Firth_selected)

# Obtain coefficient + profile penalized likelihood CIs (invalid CIs)
cOR_selected_Firth_Est <- round(exp(coef( Firth_selected)["CT"]), digits = 2)
cOR_selected_naive_Firth_Low <- round(exp(confint(Firth_selected)[2,1]),
                                      digits=2)
cOR_selected_naive_Firth_Up  <- round(exp(confint(Firth_selected)[2,2]),
                                      digits=2)
```

```
paste0(cOR_selected_Firth_Est,
       "(95% CI, ", cOR_selected_naive_Firth_Low,
       "; ", cOR_selected_naive_Firth_Up,")")


# Marginal RR, FLIC, backward elimination ----
#---------------------------------------------------------------------------------#

# Obtain predicted potential outcomes
PredA0 <- as.vector(predict(Firth_selected,
                            newdata = All_unexposed, type = "response"))
PredA1 <- as.vector(predict(Firth_selected,
                            newdata = All_exposed, type = "response"))

# Estimate Marginal RR
mRR_selected_Firth_Est <- mean(PredA1)/mean(PredA0)


# Bootstrap confidence interval ----
#---------------------------------------------------------------------------------#

b_rep <- 500
seeds <- 1:b_rep
log_cOR_full <-
  log_cOR_selected <-
    log_cOR_selected_naive <- matrix(NA,
                                     ncol = length(coef(Firth_full)),
                                     nrow = b_rep,
                                     dimnames=list(NULL,names(coef(Firth_full))))

log_mRR_full     <-
  log_mRR_selected <-
    log_mRR_selected_naive <- matrix(NA, nrow = b_rep, ncol = 1)

# Run for 500 bootstrap resamplings
for(i in 1:b_rep){

  # Sample bootstrap data using prespecified seeds
  set.seed(seeds[i])

  # Bootstrap sampling ----
  # Stratified sampling, events
  events            <- CABG_data[CABG_data$Postoperative.stroke ==1,]
  sampled_events    <- sample(1:nrow(events),
                              size = nrow(events), replace = TRUE)
  bs_events         <- events[sampled_events,]
  # Sample non-events
  nonevents         <- CABG_data[CABG_data$Postoperative.stroke == 0,]
  sampled_nonevents <- sample(1:nrow(nonevents),
                              size = nrow(nonevents), replace = TRUE)
  bs_nonevents      <- nonevents[sampled_nonevents,]

  # Complete bootstrap sample
  bs_sample         <- rbind(bs_events,bs_nonevents)

  # Remove variables with no variance from the full model
  drop <- colnames(bs_sample)[sapply(bs_sample, function(x)
    ifelse(class(x) == "factor",
           all(duplicated(x)[-1L]),
           var(x)==0))]
  bs_sample <- bs_sample[,!(colnames(bs_sample) %in% drop)]
```

```
# Create potential outcome datasets
All_unexposed_bs <- bs_sample
All_unexposed_bs$CT <- 0
All_exposed_bs <- bs_sample
All_exposed_bs$CT <- 1

# Full model ----
# Estimate Firth model in bootstrap sample
Firth_full_bs <- logistf(as.formula(paste0("Postoperative.stroke ~ ",
                                paste(colnames(bs_sample[,2:ncol(bs_sample)]),
                                collapse = "+"))),
                           data = bs_sample,
                           control = logistf.control(maxit = 200,maxstep = 5),
                           firth = TRUE, # firth = FALSE for ML estimation
                           pl = FALSE,   # no profile likelihood CIs
                           flic = TRUE)

# Store coefficients
log_cOR_full[i, names(coef(Firth_full_bs))] <- coef(Firth_full_bs)

# Obtain predicted potential outcomes
PredA0 <- as.vector(predict(Firth_full_bs,
                            newdata = All_unexposed_bs, type = "response"))
PredA1 <- as.vector(predict(Firth_full_bs,
                            newdata = All_exposed_bs, type = "response"))



# Estimate mRR in bootstrap sample
log_mRR_full[i,] <- log(mean(PredA1)/mean(PredA0))


# Selected model do not repeat selection (naive) ----
# Perform backward elimination in the bootstrap sample
select_predictors       <- all.vars(Firth_selected$formula)
Firth_selected_naive_bs <- logistf(as.formula(paste0("Postoperative.stroke~",
                                                   paste(select_predictors[
                                                   !(select_predictors %in%
                                                   c("Postoperative.stroke",
                                                     drop))],
                                                     collapse = "+"))),
                                            # take into account that covs.
                                            # may be removed from bs sample
                                   data = bs_sample,
                                   control = logistf.control(maxit = 200,
                                                             maxstep = 5),
                                   firth = TRUE,
                                   pl = FALSE,
                                   flic = TRUE)

# Store coefficients
log_cOR_selected_naive[i, names(coef(Firth_selected_naive_bs))] <-
  coef(Firth_selected_naive_bs)

# Obtain predicted potential outcomes
PredA0 <- as.vector(predict(Firth_selected_naive_bs,
                            newdata = All_unexposed_bs, type = "response"))
PredA1 <- as.vector(predict(Firth_selected_naive_bs,
                            newdata = All_exposed_bs, type = "response"))
```

```
  # Estimate mRR in bootstrap sample
  log_mRR_selected_naive[i,] <- log(mean(PredA1)/mean(PredA0))

  # Selected model repeat selection (correct) ----
  # Perform backward elimination in the bootstrap sample
  Firth_selected_bs <- backward(Firth_full_bs,
                              scope = colnames(bs_sample)[
                                !(colnames(bs_sample) %in%
                                    c("Postoperative.stroke","CT"))],
                              slstay = 0.157,
                              trace = FALSE,
                              pl = FALSE,     # waste of computational power
                              data = bs_sample,
                              control = logistf.control(maxit = 200,
                                                         maxstep = 5))

  # Re-estimate intercept in bootstrap sample
  Firth_selected_bs      <- flic(Firth_selected_bs)

  # Store coefficients
  log_cOR_selected[i, names(coef(Firth_selected_bs))] <-
    coef(Firth_selected_bs)

  # Obtain predicted potential outcomes
  PredA0 <- as.vector(predict(Firth_selected_bs,
                            newdata = All_unexposed_bs, type = "response"))
  PredA1 <- as.vector(predict(Firth_selected_bs,
                            newdata = All_exposed_bs, type = "response"))

  # Estimate mRR in bootstrap sample
  log_mRR_selected[i,] <- log(mean(PredA1)/mean(PredA0))


}


# Summarize results
# Obtain valid cOR CIs
cOR_selected_Firth_Low <- round(quantile(exp(log_cOR_selected), 0.025),
                                digits = 2)
cOR_selected_Firth_Up  <- round(quantile(exp(log_cOR_selected), 0.975),
                                digits = 2)
paste0(round(cOR_selected_Firth_Est,digits=2),
       "(95% CI, ", cOR_selected_Firth_Low,
       "; ", cOR_selected_Firth_Up,")")

# Obtain mRR + CI
mRR_full_Firth_Low <- round(quantile(exp(log_mRR_full), 0.025, na.rm = T),
                            digits = 2)
mRR_full_Firth_Up  <- round(quantile(exp(log_mRR_full), 0.975, na.rm = T),
                            digits = 2)
paste0(round(mRR_full_Firth_Est,digits=2),
       "(95% CI, ", mRR_full_Firth_Low,
       "; ", mRR_full_Firth_Up,")")

# Obtain mRR + CI model with backward elimination
mRR_selected_Firth_Low <- round(quantile(exp(log_mRR_selected), 0.025),
                                digits = 2)
mRR_selected_Firth_Up  <- round(quantile(exp(log_mRR_selected), 0.975),
                                digits = 2)
```

```
paste0(round(mRR_selected_Firth_Est,digits=2),
       "(95% CI, ", mRR_selected_Firth_Low,
       "; ", mRR_selected_Firth_Up,")")


# Relative Conditional Bias and Root Mean Squared Difference Ratio ----
#------------------------------------------------------------------------------#

# Stability measure: selection frequencies full model
boot_01_full <- (log_cOR_full != 0) * 1
boot_inclusion_full <- apply(boot_01_full, 2, function(x)
  sum(x, na.rm=T)/length(x)*100)

# Stability measure: selection frequencies backward eliminated model
boot_01_selected <- (log_cOR_selected != 0) * 1
boot_inclusion_selected <- apply(boot_01_selected, 2,
                                 function(x) sum(x, na.rm=T)/length(x)*100)

# Conditional OR ----

## Visualize the distributions of log conditional OR
type_boot <- c(rep("full",500), rep("selected",500), rep("selected-naive", 500))
log_cOR   <- c(log_cOR_full, log_cOR_selected, log_cOR_selected_naive)

ggplot(NULL, aes(log_cOR,fill=type_boot)) + geom_density()

## Relative conditional bias
cat("RCB (%):\n")
(RCB_log_cOR <- (mean(log_cOR_selected)/Firth_full)-1)*100

## Root mean squared difference ratio
RMSD_log_cOR    <- sqrt(mean((log_cOR_selected - Firth_full)**2))
se_full_log_cOR <- sd(log_cOR_full)
cat("RMSDR:\n")
(RMSDR_log_cOR  <- RMSD_log_cOR/se_full_log_cOR)

## Illustration that not repeating selection underestimates standard error
cat("Standard error full:\n")
sqrt(mean((log_cOR_full - Firth_full)**2))
cat("Standard error selected:\n")
sqrt(mean((log_cOR_selected - Firth_selected)**2))
cat("Standard error selected-naive:\n")
sqrt(mean((log_cOR_selected_naive - Firth_selected)**2))


# Marginal RR ----

## Visualize the distributions of log marginal RR
type_boot <- c(rep("full",500), rep("selected",500), rep("selected-naive", 500))
log_mRR   <- c(log_mRR_full, log_mRR_selected, log_mRR_selected_naive)

ggplot(NULL, aes(log_mRR,fill = type_boot)) + geom_density()

## Relative conditional bias
cat("RCB (%):\n")
(RCB_log_mRR <- (mean(log_mRR_selected)/Firth_full)-1)*100

## Root mean squared difference ratio
RMSD_log_mRR    <- sqrt(mean((log_mRR_selected - Firth_full)**2))
se_full_log_MRR <- sd(log_mRR_full)
cat("RMSDR:\n")
```

```
(RMSDR_log_mRR  <- RMSD_log_mRR/se_full_log_MRR)

## Illustration that not repeating selection underestimates standard error
cat("Standard error full:\n")
sqrt(mean((log_mRR_full - Firth_full)**2))
cat("Standard error selected:\n")
sqrt(mean((log_mRR_selected - Firth_selected)**2))
cat("Standard error selected-naive:\n")
sqrt(mean((log_mRR_selected_naive - Firth_selected)**2))
```
