# Supplementary material for: A comparison of full model specification and backward elimination of potential confounders when estimating marginal and conditional causal effects on binary outcomes from observational data
Source: Biom J. 2022 May 12;66(1):2100237. doi: 10.1002/bimj.202100237 (PMC10952199; doi:10.1002/bimj.202100237)
Supplement: Supplementary file 2 — Supporting information [file BIMJ-66-0-s003.pdf]

Supplementary File 2

Mean squared error of ordinary least squares exposure effect estimator of full and reduced model

This file describes analytical expressions for the mean squared error (MSE) of an exposure effect estimator in a linear model, referred to in section 2.1 in the main text of "A comparison of full model specification and backward elimination of potential confounders when estimating marginal and conditional causal effects on binary outcomes from observational data", by Kim Luijken, Rolf H.H. Groenwold, Maarten van Smeden, Susanne Strohmaier, and Georg Heinze.

Notation and set-up

Consider the model depicted in Figure 1. Let A denote the exposure, L a covariate and Y the outcome. Each variable is a linear combination of the variables affecting it (indicated by the directed arrows in Figure 1) plus an error term. The coefficients of the model are denoted α for the relation between A and L, γ for the relation between Y and L conditional on A and β for the relation between Y and A conditional on L. All variables are normally distributed, where $L \sim \mathcal{N}(\mu_L, \sigma_L^2)$, $A \sim \mathcal{N}(\mu_A, \sigma_A^2)$, and $Y \sim \mathcal{N}(\mu_Y, \sigma_Y^2)$. We estimate the model using ordinary least squares regression, where we assume all errors to be uncorrelated, no misspecification of functional forms, no measurement error, no missing data and no interactions.

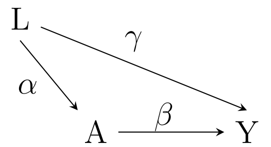

Figure 1: Directed acyclic graph

To explore when elimination of a covariate improves efficiency of an ordinary least squares estimator of

the association between exposure and the outcome (in short exposure effect estimator), we derive expressions for the MSE of the regression coefficient of Y on A when L is included in the model (full model), $\text{MSE}(\hat{\beta})$, and for the model where L is omitted (reduced model), $\text{MSE}(\hat{\beta}_{omit})$. The expression for bias of the exposure effect estimator is derived using path-tracing rules, as described by Wright [1] and Pearl [2]. The variance of the exposure effect estimator is specified for an ordinary least squares estimator in a finite sample with n observations, similar to the expressions for the extended omitted variable framework by Cinelli and Hazlett [3].

Full model

Consider an ordinary least squares regression of the model in Figure 1,

$$Y = \hat{\beta}A + \hat{\gamma}L + \hat{\epsilon},$$

where Y is an $n \times 1$ vector containing the outcome of interest for each of the n observations, A is an $n \times 1$ vector of the continuous exposure variable, L is an $n \times 1$ vector of the continuous covariate, $\hat{\beta}$ and $\hat{\gamma}$ are ordinary least squares coefficients of the association between Y and A and between Y and L, respectively, and $\epsilon \sim \mathcal{N}(0, \sigma^2_{Y^{\perp A,L}})$. Let $Y^{\perp A,L}$ denote the variable Y after removing the components linearly explained by A and L, $A^{\perp L}$ denote the variable A after removing the components linearly explained by L, $\hat{\alpha}$ denote the ordinary least squares estimator of the association between A and L and $\text{var}(\cdot)$ and $\text{cov}(\cdot)$ denote the sample variances and covariances, respectively [3]. Then, using path-tracing rules [1, 2]

$$\begin{aligned}
\text{var}(L) &:= \hat{\sigma}^2_L \\
\text{var}(A) &:= \hat{\sigma}^2_A \\
\text{cov}(A, L) &:= \hat{\sigma}^2_L\hat{\alpha} \\
\text{var}(A^{\perp L}) &:= \hat{\sigma}^2_A - \hat{\sigma}^2_L\hat{\alpha}^2 \\
\text{var}(Y) &:= \hat{\sigma}^2_Y \\
\text{cov}(Y^{\perp L}, A^{\perp L}) &:= (\hat{\sigma}^2_A - \hat{\sigma}^2_L\hat{\alpha}^2)\hat{\beta} \\
\text{var}(Y^{\perp A,L}) = \hat{\sigma}^2_{Y^{\perp A,L}} &:= \hat{\sigma}^2_Y - \hat{\sigma}^2_A\hat{\beta}^2 - \hat{\sigma}^2_L\hat{\gamma}^2 - 2\hat{\sigma}^2_L\hat{\alpha}\hat{\beta}\hat{\gamma}.
\end{aligned}$$

As described in [1, 2], partial regression coefficients can be readily read from a path diagram such as Figure 1. The expected value for the partial regression coefficient regressing Y on A given L, $\hat{\beta}$, can be

expressed as

$$\hat{\beta} = \frac{\text{cov}(Y^{\perp L}, A^{\perp L})}{\text{var}(A^{\perp L})}, \text{ and}$$
$$\begin{aligned}\mathbb{E}(\hat{\beta}) &= \frac{(\sigma_A^2 - \sigma_L^2\alpha^2)\beta}{\sigma_A^2 - \sigma_L^2\alpha^2} \\ &= \beta. \end{aligned} \tag{1}$$

Hence, the bias of the effect estimator in the full model is $\mathbb{E}(\hat{\beta}) - \beta = 0$.

Let df denote the ordinary least squares regression's degrees of freedom. An expression for the variance of $\hat{\beta}$ is [3]

$$\begin{aligned}\text{var}(\hat{\beta}) &= \frac{\text{var}(Y^{\perp A,L})}{\text{var}(A^{\perp L})}\frac{1}{\text{df}-1} \\ &= \frac{\hat{\sigma}_Y^2 - \hat{\sigma}_A^2\hat{\beta}^2 - \hat{\sigma}_L^2\hat{\gamma}^2 - 2\hat{\sigma}_L^2\hat{\alpha}\hat{\beta}\hat{\gamma}}{\hat{\sigma}_A^2 - \hat{\sigma}_L^2\hat{\alpha}^2}\frac{1}{n-3}.\end{aligned}$$

To derive the expected variance of $\hat{\beta}$, we use the property that $\hat{\sigma}^2 \approx \hat{\sigma}^2\frac{n-1}{n} \approx \sigma^2$ for $\hat{\sigma}_A^2$, $\hat{\sigma}_L^2$ and $\hat{\sigma}_Y^2$. Furthermore, we assume that the residuals $\hat{\sigma}^2_{Y^{\perp A,L}}$ are independent from A and L and apply a first-order approximation [4]. Then,

$$\mathbb{E}[\text{var}(\hat{\beta})] \approx \frac{\sigma_Y^2 - \sigma_A^2\beta^2 - \sigma_L^2\gamma^2 - 2\sigma_L^2\alpha\beta\gamma}{\sigma_A^2 - \sigma_L^2\alpha^2}\frac{1}{n-3}. \tag{2}$$

Since $MSE = bias^2 + variance$,

$$MSE(\hat{\beta}) \approx \frac{\sigma_Y^2 - \sigma_A^2\beta^2 - \sigma_L^2\gamma^2 - 2\sigma_L^2\alpha\beta\gamma}{\sigma_A^2 - \sigma_L^2\alpha^2}\frac{1}{n-3}. \tag{3}$$

.

Reduced model

Consider the ordinary least squares regression of the model in Figure 1 where variable L is omitted

$$Y = \hat{\beta}_{omit}A + \hat{\epsilon}_{omit},$$

where Y is an $n \times 1$ vector containing the outcome of interest for each of the n observations, A is an $n \times 1$ continuous exposure variable, $\hat{\beta}_{omit}$ is an ordinary least squares coefficient estimator and $\epsilon_{omit} \sim \mathcal{N}(0, \sigma^2_{Y^{\perp A}})$.

Let $Y^{\perp A}$ denote the variable Y after removing the components linearly explained by A. Then,

$$\begin{aligned}\text{cov}(Y, A) &:= \hat{\sigma}_A^2 \hat{\beta} + \hat{\sigma}_L^2 \hat{\alpha} \hat{\gamma} \\ \text{var}(Y^{\perp A}) = \hat{\sigma}_{Y^{\perp A}}^2 &:= \hat{\sigma}_Y^2 - \hat{\sigma}_A^2 \hat{\beta}^2 - 2\hat{\sigma}_L^2 \hat{\alpha} \hat{\beta} \hat{\gamma}.\end{aligned}$$

Again, reading partial regressions from Figure 1 as described in [1, 2], we find that the expected value for the marginal association between A and Y can be expressed as

$$\begin{aligned}\hat{\beta}_{omit} &= \frac{\text{cov}(Y, A)}{\text{var}(A)}, \text{ and} \\ \mathbb{E}(\hat{\beta}_{omit}) &= \frac{\sigma_A^2 \beta + \sigma_L^2 \alpha \gamma}{\sigma_A^2} \\ &= \beta + \frac{\sigma_L^2 \alpha \gamma}{\sigma_A^2}. \end{aligned} \tag{4}$$

Hence, the expected bias in the reduced model is $\mathbb{E}(\hat{\beta}_{omit}) - \beta = \frac{\sigma_L^2 \alpha \gamma}{\sigma_A^2}$. We obtain the expression for the variance of $\hat{\beta}_{omit}$ [3]

$$\begin{aligned}\text{var}(\hat{\beta}_{omit}) &= \frac{\text{var}(Y^{\perp A})}{\text{var}(A)} \frac{1}{\text{df}} \\ &= \frac{\hat{\sigma}_Y^2 - \hat{\sigma}_A^2 \hat{\beta}^2 - 2\hat{\sigma}_L^2 \hat{\alpha} \hat{\beta} \hat{\gamma}}{\hat{\sigma}_A^2} \frac{1}{n-2}.\end{aligned}$$

To derive the expected variance of $\hat{\beta}_{omit}$, we use the property that $\hat{\sigma}^2 \approx \hat{\sigma}^2 \frac{n-1}{n} \approx \sigma^2$ for $\hat{\sigma}_A^2$ and $\hat{\sigma}_Y^2$. Furthermore, we assume that the residuals $\hat{\sigma}_{Y^{\perp A}}^2$ are independent from A and apply a first-order approximation [4]. Then,

$$\mathbb{E}[\text{var}(\hat{\beta}_{omit})] \approx \frac{\sigma_Y^2 - \sigma_A^2 \beta^2 - 2\sigma_L^2 \alpha \beta \gamma}{\sigma_A^2} \frac{1}{n-2}, \tag{5}$$

where the final step is performed using a first-order approximation [4] and under the assumption that the

residuals $\hat{\sigma}^2_{Y \perp A}$ are independent from A. Since $MSE = bias^2 + variance$,

$$
\begin{aligned}
MSE(\hat{\beta}_{omit}) &\approx \left(\frac{\sigma_L^2 \alpha\gamma}{\sigma_A^2}\right)^2 + \frac{\sigma_Y^2 - \sigma_A^2\beta^2 - 2\sigma_L^2\alpha\beta\gamma}{\sigma_A^2}\frac{1}{n-2} \\
&\approx \frac{\sigma_L^4\alpha^2\gamma^2}{\sigma_A^4} + \frac{\sigma_Y^2 - \sigma_A^2\beta^2 - 2\sigma_L^2\alpha\beta\gamma}{\sigma_A^2}\frac{1}{n-2}. \qquad (6)
\end{aligned}
$$

Comparison full and reduced model

Plotting equation (3) and equation (6) for the arbitrarily chosen values $\alpha = 0.4$, $\beta = 0.3$, $\gamma = 0.2$, $\sigma_A^2 = 2.5$, $\sigma_L^2 = 8$, and $\sigma_Y^2 = 10$ yields the following result.

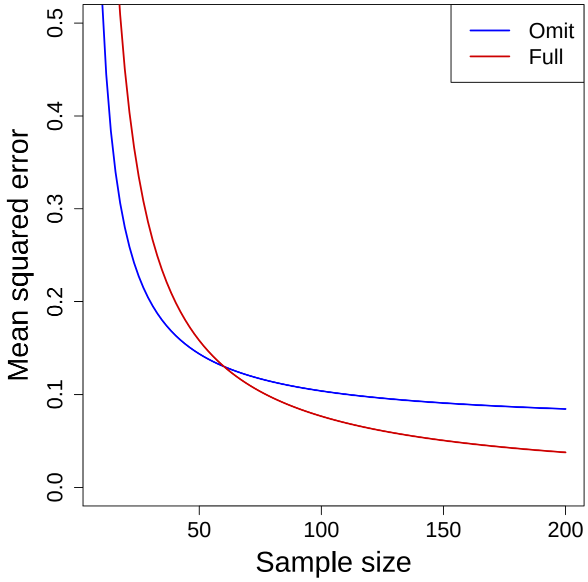

Figure 2: Illustration of the bias-variance trade-off for the ordinary least squares estimator of the exposure effect when including (Full) or omitting (Omit) covariate L. The blue and red line are computed using expression (6) and (3), respectively, for sample size n ranging from 0 to 200 and the values $\alpha = 0.4$, $\beta = 0.3$, $\gamma = 0.2$, $\sigma_A^2 = 2.5$, $\sigma_L^2 = 8$, and $\sigma_Y^2 = 10$. The value of n for which the reduced variance by omitting L outweighs the increase in squared bias is around 60.

The lines in Figure 2 show that $MSE(\hat{\beta}_{omit}) < MSE(\hat{\beta})$ for smaller sample size n, and that the full model has a lower MSE for $\hat{\beta}$ with larger sample sizes. In other words, if the inequality $Bias^2_{omit} < \text{var}(\hat{\beta}) - \text{var}(\hat{\beta}_{omit})$ holds, i.e., when

$$
\frac{\sigma_L^4\alpha^2\gamma^2}{\sigma_A^4} < \frac{\sigma_Y^2 - \sigma_A^2\beta^2 - \sigma_L^2\gamma^2 - 2\sigma_L^2\alpha\beta\gamma}{\sigma_A^2 - \sigma_L^2\alpha^2}\frac{1}{n-3} - \frac{\sigma_Y^2 - \sigma_A^2\beta^2 - 2\sigma_L^2\alpha\beta\gamma}{\sigma_A^2}\frac{1}{n-2}
$$

holds, then the reduced variance by omitting L outweighs the increase in squared bias. A simple approxi-

mation of the value of n for which this is the case, denoted n', can be found by assuming $n-3 \approx n-2 \approx n$ (which is reasonable to assume for sufficiently large sample sizes). This yields the following expression

$$n' < \frac{\sigma_A^4\sigma_Y^2 - \sigma_A^6\beta^2 - \sigma_A^4\sigma_L^2\gamma^2 - 2\sigma_A^4\sigma_L^2\alpha\beta\gamma}{(\sigma_A^2 - \sigma_L^2\alpha^2)\sigma_L^4\alpha^2\gamma^2} - \frac{\sigma_A^2\sigma_Y^2 - \sigma_A^4\beta^2 - 2\sigma_A^2\sigma_L^2\alpha\beta\gamma}{\sigma_L^4\alpha^2\gamma^2}. \tag{7}$$

Similar to the plot above, Equation (7) indicates that for sample sizes smaller than the critical n', omitting L results in a lower mean squared error of the exposure estimate than including it. The impact of many of the parameters in the equation depends on the values of other parameters. Their joint effect on the critical sample size could be assessed by means of plotting the result of equation for various parameters values, as is presented in Figures 3 and 4.

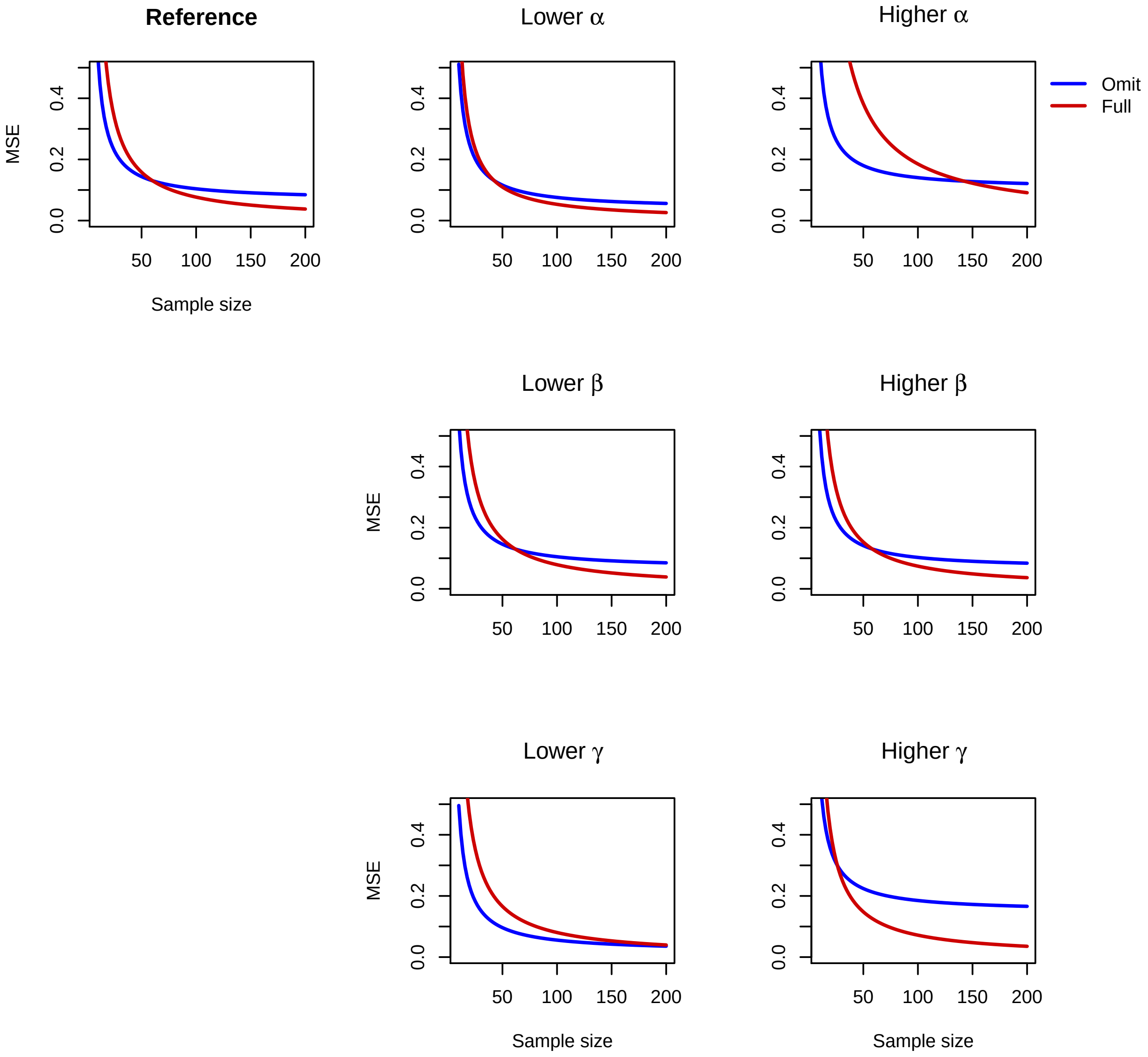

Figure 3: Illustration of the bias-variance trade-off for the ordinary least squares estimator of the exposure effect when including (Full) or omitting (Omit) covariate L. The blue and red line are computed using expression (6) and (3), respectively, for sample size n ranging from 0 to 200. The parameters σ_A^2, σ_L^2, and σ_Y^2 are fixed to values 2.5, 8 and 10, respectively, while the values of parameters α, β, and γ are varied. The reference plot is created using the arbitrarily chosen values $\alpha = 0.4$, $\beta = 0.3$, $\gamma = 0.2$, $\sigma_A^2 = 2.5$, $\sigma_L^2 = 8$, and $\sigma_Y^2 = 10$. Lower α indicates $\alpha = 0.3$ and higher $\alpha = 0.5$. Lower β indicates $\beta = 0.2$ and higher $\beta = 0.4$. Lower γ indicates $\gamma = 0.1$ and higher $\gamma = 0.3$

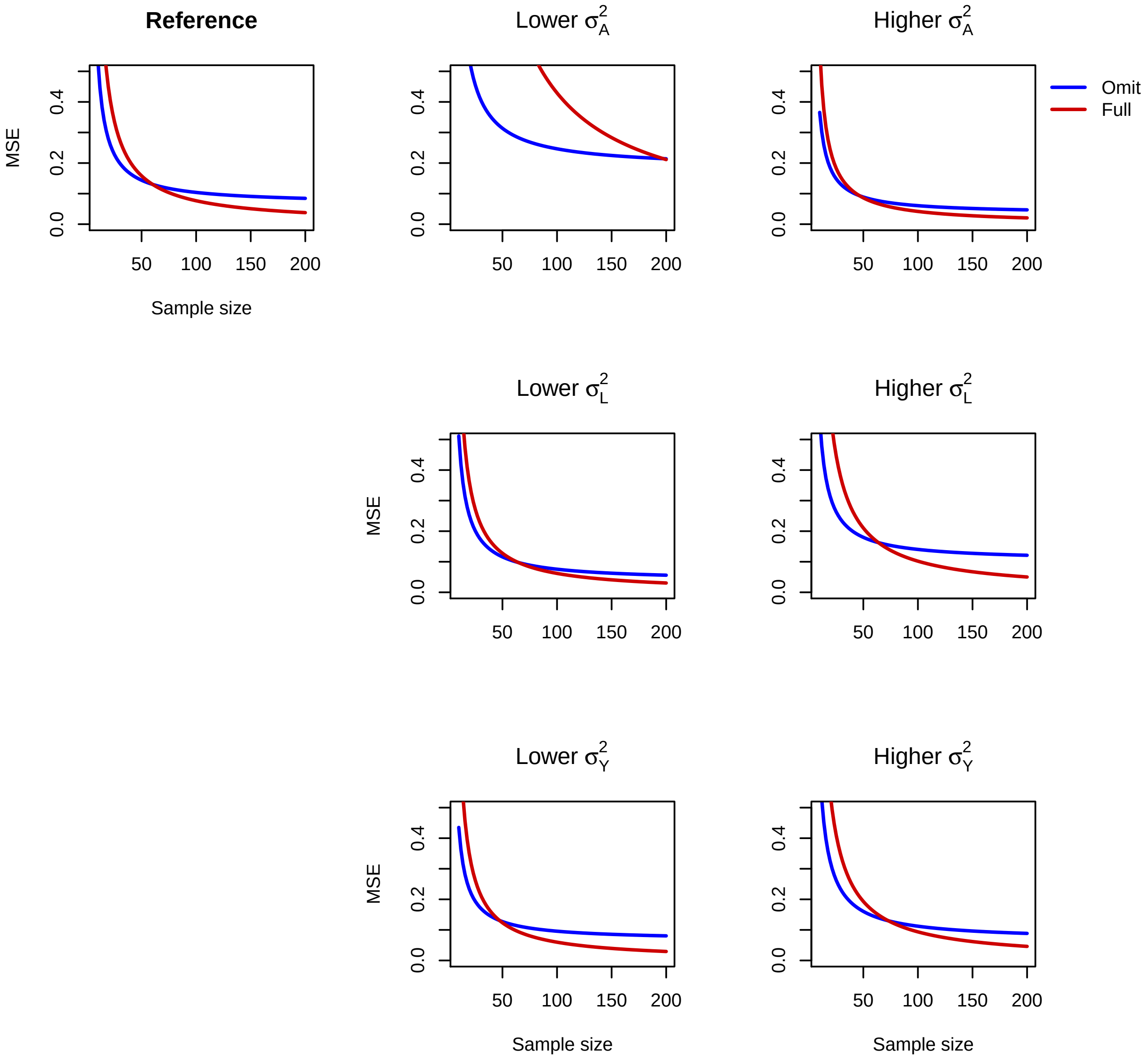

Figure 4: Illustration of the bias-variance trade-off for the ordinary least squares estimator of the exposure effect when including (Full) or omitting (Omit) covariate L. The blue and red line are computed using expression (6) and (3), respectively, for sample size n ranging from 0 to 200. The parameters α, β, and γ are fixed to values 0.4, 0.3 and 0.2, respectively, while the values of parameters σ_A^2, σ_L^2, and σ_Y^2 are varied. The reference plot is created using the arbitrarily chosen values $\alpha = 0.4$, $\beta = 0.3$, $\gamma = 0.2$, $\sigma_A^2 = 2.5$, $\sigma_L^2 = 8$, and $\sigma_Y^2 = 10$. Lower σ_A^2 indicates $\sigma_A^2 = 1.5$ and higher $\sigma_A^2 = 3.5$. Lower σ_L^2 indicates $\sigma_L^2 = 6$ and higher $\sigma_L^2 = 10$. Lower σ_Y^2 indicates $\sigma_Y^2 = 8$ and higher $\sigma_Y^2 = 12$.

References

[1] S. Wright, Correlation and causation, J. agric. Res. 20 (1921) 557–580.

[2] J. Pearl, Linear models: A useful "microscope" for causal analysis, Journal of Causal Inference 1 (1) (2013) 155–170.

[3] C. Cinelli, C. Hazlett, Making sense of sensitivity: Extending omitted variable bias, Journal of the Royal Statistical Society: Series B (Statistical Methodology) 82 (1) (2020) 39–67.

[4] L. Henckel, E. Perković, M. H. Maathuis, Graphical criteria for efficient total effect estimation via adjustment in causal linear models, arXiv preprint arXiv:1907.02435 (2019).
