# Supplementary material for: A comparison of full model specification and backward elimination of potential confounders when estimating marginal and conditional causal effects on binary outcomes from observational data
Source: Biom J. 2022 May 12;66(1):2100237. doi: 10.1002/bimj.202100237 (PMC10952199; doi:10.1002/bimj.202100237)
Supplement: Supplementary file 3 — Supporting information [file BIMJ-66-0-s005.pdf]

Supplementary File 3

Simulation experiment 1, results

This file provides the results of the proof-of-concept simulations (simulation experiment 1), referred to in section 3 and 4 in the main text of “A comparison of full model specification and backward elimination of potential confounders when estimating marginal and conditional causal effects on binary outcomes from observational data”, by Kim Luijken, Rolf H.H. Groenwold, Maarten van Smeden, Susanne Strohmaier, and Georg Heinze.

We present results for the conditional odds ratio (cOR) using Firth’s Logistic regression with Intercept Correction (FLIC) and results for the cOR and marginal risk ratio (mRR) estimated using Maximum Likelihood (ML) estimation. Figures in this supplementary file show the trade-off in $bias^2$ and *variance* for simulated data, illustrating principle (1) in the main text. A single covariate L acts as a noise variable, (near-)instrumental variable, confounder, or (near-)predictor in a setting where a binary outcome has a true null effect on a binary outcome. All plots were created using the `looplot` package in R.

Conditional OR, FLIC

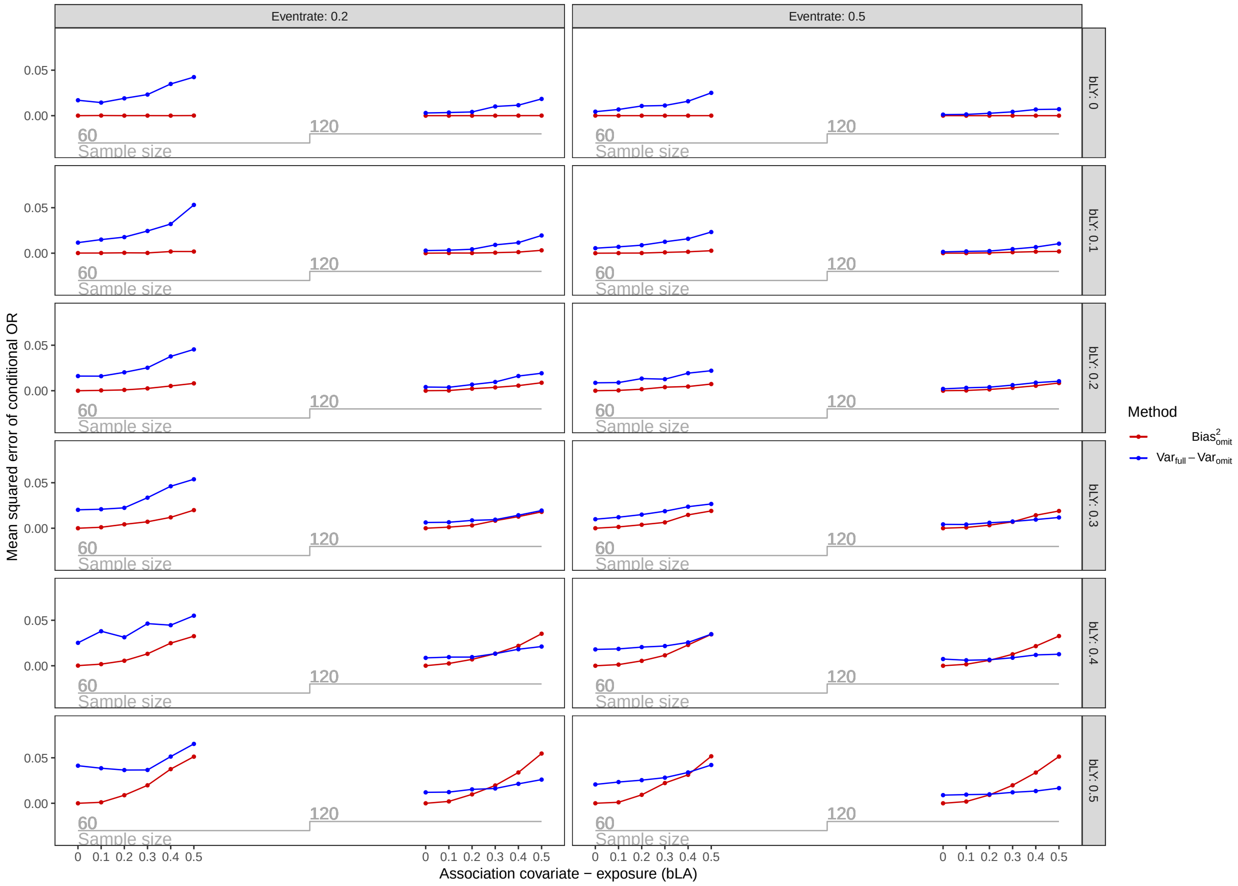

Figure 1: Results of simulation experiment 1 for the logarithm of the cOR using FLIC estimation.

Conditional OR, ML

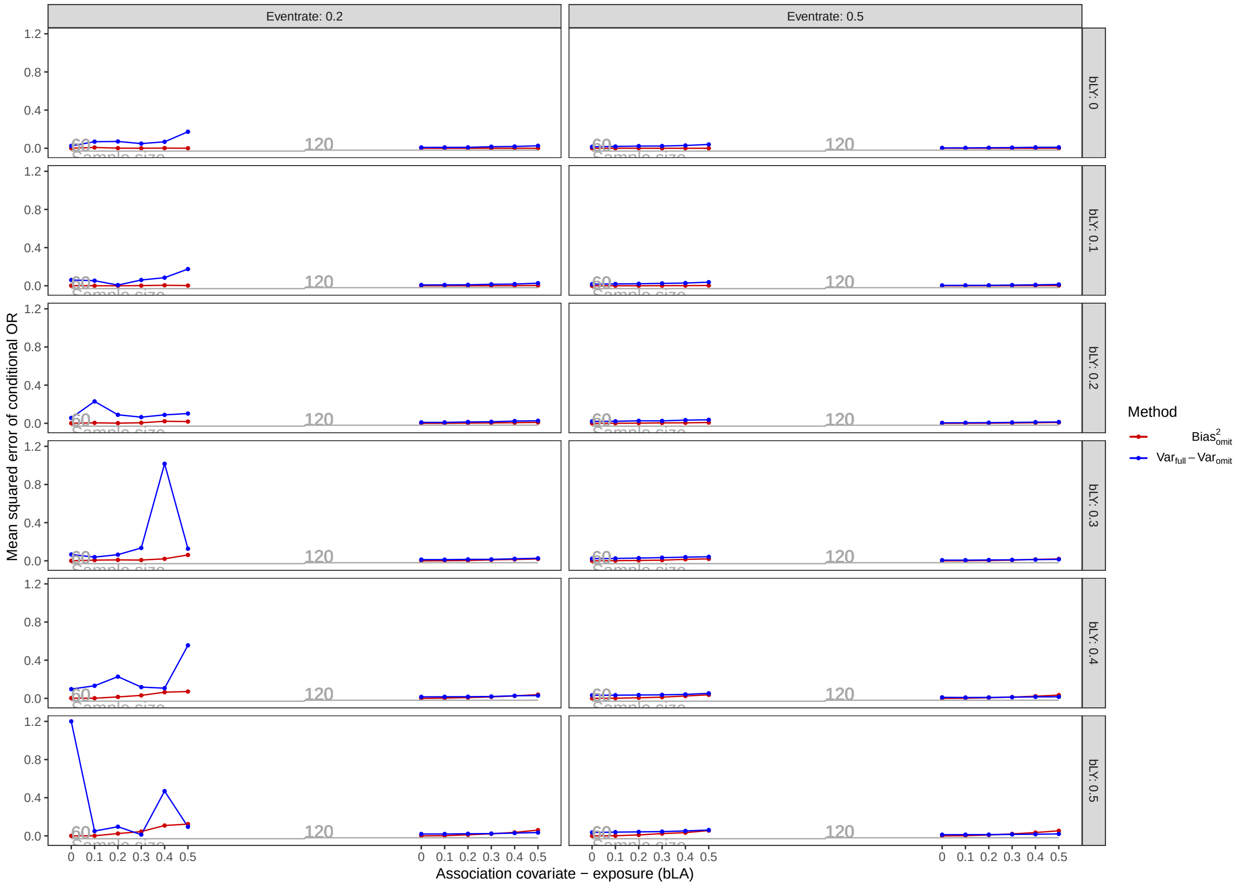

Figure 2: Results of simulation experiment 1 for the logarithm of the cOR using ML estimation.

Marginal RR, ML

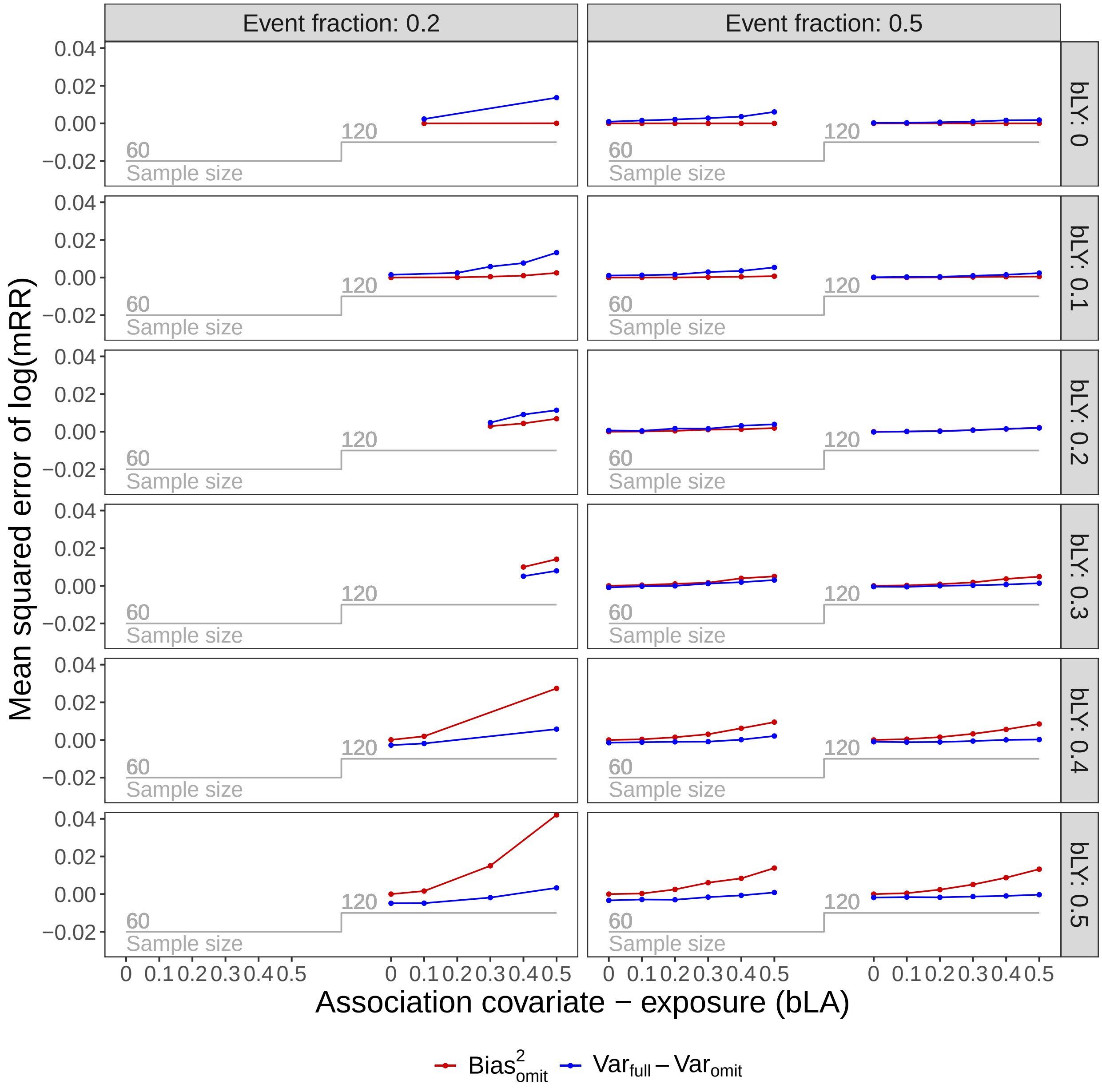

Figure 3: Results of simulation experiment 1 for the logarithm of the mRR using ML estimation. In 53 out of 144 scenarios, the MRR could not be estimated using Maximum Likelihood estimation, due to convergence issues.
