# Supplementary material for: A comparison of full model specification and backward elimination of potential confounders when estimating marginal and conditional causal effects on binary outcomes from observational data
Source: Biom J. 2022 May 12;66(1):2100237. doi: 10.1002/bimj.202100237 (PMC10952199; doi:10.1002/bimj.202100237)

Supplementary File 4

Simulation experiment 2, results

This file provides the results of simulation experiment 2, referred to in section 3 and 4 in the main text of "A comparison of full model specification and backward elimination of potential confounders when estimating marginal and conditional causal effects on binary outcomes from observational data", by Kim Luijken, Rolf H.H. Groenwold, Maarten van Smeden, Susanne Strohmaier, and Georg Heinze. To facilitate replicability of simulation results, we present some additional descriptives of the simulation ouput.

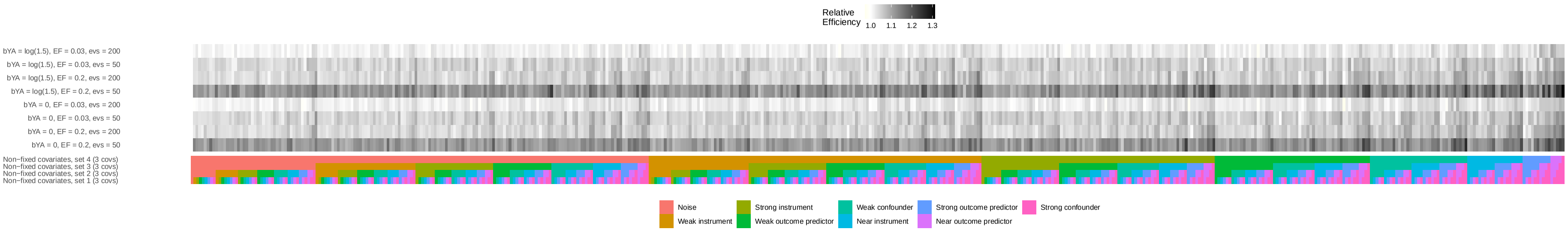

Figure 1: The relative efficiency of the marginal risk ratio estimated using Firth's corrected Logistic regression with intercept-correction between the full and selected model in each of the 3960 scenarios. The relative efficiency is computed as $\frac{MSE_{selected}}{MSE_{full}}$

Firth's corrected Logistic regression with intercept-correction

Proportion of true confounders eliminated

			Proportion of true confounders eliminated		
bYA	event fraction	nevents	Full	Selected	Unadj.
0.00	0.20	50	0.00	0.75	1.00
0.00	0.20	200	0.00	0.73	1.00
0.00	0.03	50	0.00	0.75	1.00
0.00	0.03	200	0.00	0.72	1.00
log(1.5)	0.20	50	0.00	0.75	1.00
log(1.5)	0.20	200	0.00	0.73	1.00
log(1.5)	0.03	50	0.00	0.74	1.00
log(1.5)	0.03	200	0.00	0.72	1.00

Table 1: The proportion of true confounders eliminated by backward elimination in FLIC estimation.
Each row represents 495 scenarios with varying associations between the covariates and the exposure and/or outcome.

Marginal Risk Ratio, Firth's corrected Logistic regression with intercept-correction

Values

				Mean			Min			Max			Warnings	
bYA	event fraction	nevents	Full	Selected	Unadj.	Full	Selected	Unadj.	Full	Selected	Unadj.	Full	Selected	Unadj.
0.00	0.20	50	0.09	0.12	0.59	-0.02	-0.00	0.09	0.31	0.37	1.46	0	0	0
0.00	0.20	200	0.10	0.12	0.61	-0.02	-0.01	0.16	0.32	0.34	1.66	0	0	0
0.00	0.03	50	0.13	0.15	0.67	-0.02	-0.00	0.11	0.41	0.47	2.02	0	0	0
0.00	0.03	200	0.13	0.15	0.65	-0.03	-0.01	0.16	0.41	0.44	1.60	0	0	0
log(1.5)	0.20	50	0.22	0.25	0.70	-0.01	0.00	0.15	0.41	0.45	1.57	0	0	0
log(1.5)	0.20	200	0.22	0.24	0.72	-0.01	-0.00	0.09	0.41	0.45	1.64	0	0	0
log(1.5)	0.03	50	0.22	0.25	0.78	-0.03	0.00	0.19	0.40	0.43	1.65	0	0	0
log(1.5)	0.03	200	0.22	0.24	0.77	-0.01	0.00	0.11	0.40	0.44	2.04	0	0	0

Table 2: The values of the log(mRR) estimated using FLIC. Each row represents 495 scenarios with varying associations between the covariates and the exposure and/or outcome.

Bias

				Mean			Min			Max			Warnings	
bYA	event fraction	nevents	Full	Selected	Unadj.	Full	Selected	Unadj.	Full	Selected	Unadj.	Full	Selected	Unadj.
0.00	0.20	50	-0.00	0.03	0.49	-0.04	-0.00	0.09	0.02	0.10	1.27	0	0	0
0.00	0.20	200	-0.00	0.02	0.51	-0.02	-0.01	0.10	0.03	0.07	1.66	0	0	0
0.00	0.03	50	-0.00	0.03	0.54	-0.04	-0.00	0.11	0.03	0.09	1.67	0	0	0
0.00	0.03	200	0.00	0.02	0.52	-0.03	-0.01	0.12	0.02	0.08	1.28	0	0	0
log(1.5)	0.20	50	-0.00	0.03	0.48	-0.04	-0.01	0.12	0.03	0.09	1.20	0	0	0
log(1.5)	0.20	200	-0.00	0.02	0.49	-0.04	-0.01	0.09	0.02	0.08	1.28	0	0	0
log(1.5)	0.03	50	-0.01	0.03	0.56	-0.04	-0.01	0.09	0.03	0.09	1.64	0	0	0
log(1.5)	0.03	200	-0.01	0.02	0.54	-0.04	-0.01	0.11	0.01	0.07	1.69	0	0	0

Table 3: The bias of the log(mRR) estimated using FLIC. Each row represents 495 scenarios with varying associations between the covariates and the exposure and/or outcome.

Mean Squared Error

				Mean			Min			Max			Warnings	
bYA	event fraction	nevents	Full	Selected	Unadj.	Full	Selected	Unadj.	Full	Selected	Unadj.	Full	Selected	Unadj.
0.00	0.20	50	0.06	0.06	0.35	0.02	0.02	0.06	0.10	0.12	1.65	0	0	0
0.00	0.20	200	0.04	0.04	0.36	0.01	0.01	0.03	0.08	0.09	2.78	0	0	0
0.00	0.03	50	0.08	0.08	0.43	0.01	0.01	0.04	0.20	0.21	3.01	0	0	0
0.00	0.03	200	0.04	0.05	0.36	0.01	0.01	0.03	0.11	0.13	1.74	0	0	0
log(1.5)	0.20	50	0.06	0.07	0.33	0.01	0.01	0.04	0.15	0.16	1.59	0	0	0
log(1.5)	0.20	200	0.05	0.05	0.34	0.01	0.01	0.02	0.15	0.16	1.78	0	0	0
log(1.5)	0.03	50	0.07	0.07	0.45	0.02	0.02	0.07	0.14	0.16	2.85	0	0	0
log(1.5)	0.03	200	0.04	0.04	0.41	0.02	0.02	0.03	0.10	0.11	2.89	0	0	0

Table 4: The mean squared error (MSE) of the log(mRR) estimated using FLIC. Each row represents 495 scenarios with varying associations between the covariates and the exposure and/or outcome.

Marginal Odds Ratio, Firth's corrected Logistic regression with intercept-correction

Values

				Mean			Min			Max			Warnings	
bYA	event fraction	nevents	Full	Selected	Unadj.	Full	Selected	Unadj.	Full	Selected	Unadj.	Full	Selected	Unadj.
0.00	0.20	50	0.11	0.15	0.69	-0.02	-0.01	0.12	0.38	0.45	1.77	0	0	0
0.00	0.20	200	0.12	0.15	0.70	-0.02	-0.01	0.20	0.40	0.42	1.70	0	0	0
0.00	0.03	50	0.13	0.16	0.72	-0.02	-0.00	0.12	0.42	0.48	2.07	0	0	0
0.00	0.03	200	0.13	0.16	0.71	-0.03	-0.01	0.20	0.42	0.45	1.64	0	0	0
log(1.5)	0.20	50	0.25	0.28	0.81	-0.02	0.00	0.19	0.42	0.46	1.61	0	0	0
log(1.5)	0.20	200	0.24	0.27	0.82	-0.01	-0.00	0.11	0.43	0.46	1.76	0	0	0
log(1.5)	0.03	50	0.25	0.28	0.85	-0.03	0.00	0.20	0.42	0.45	1.69	0	0	0
log(1.5)	0.03	200	0.24	0.27	0.84	-0.01	0.00	0.11	0.41	0.46	2.08	0	0	0

Table 5: The values of the log(mOR) estimated using FLIC. Each row represents 495 scenarios with varying associations between the covariates and the exposure and/or outcome.

Bias

				Mean			Min			Max			Warnings	
bYA	event fraction	nevents	Full	Selected	Unadj.	Full	Selected	Unadj.	Full	Selected	Unadj.	Full	Selected	Unadj.
0.00	0.20	50	-0.01	0.03	0.57	-0.05	-0.01	0.12	0.03	0.12	1.47	0	0	0
0.00	0.20	200	-0.00	0.02	0.58	-0.02	-0.01	0.12	0.04	0.08	1.70	0	0	0
0.00	0.03	50	-0.00	0.03	0.59	-0.04	-0.00	0.12	0.03	0.09	1.71	0	0	0
0.00	0.03	200	0.00	0.02	0.58	-0.03	-0.01	0.12	0.02	0.08	1.43	0	0	0
log(1.5)	0.20	50	-0.00	0.03	0.56	-0.04	-0.01	0.12	0.03	0.11	1.40	0	0	0
log(1.5)	0.20	200	-0.00	0.02	0.57	-0.04	-0.01	0.11	0.02	0.08	1.46	0	0	0
log(1.5)	0.03	50	-0.01	0.03	0.59	-0.05	-0.02	0.11	0.03	0.09	1.69	0	0	0
log(1.5)	0.03	200	-0.01	0.02	0.59	-0.05	-0.01	0.11	0.01	0.07	1.72	0	0	0

Table 6: The bias of the log(mOR) estimated using FLIC. Each row represents 495 scenarios with varying associations between the covariates and the exposure and/or outcome.

Mean Squared Error

				Mean			Min			Max			Warnings	
bYA	event fraction	nevents	Full	Selected	Unadj.	Full	Selected	Unadj.	Full	Selected	Unadj.	Full	Selected	Unadj.
0.00	0.20	50	0.08	0.09	0.47	0.02	0.02	0.07	0.15	0.17	2.32	0	0	0
0.00	0.20	200	0.05	0.06	0.45	0.02	0.02	0.04	0.13	0.14	2.92	0	0	0
0.00	0.03	50	0.08	0.09	0.49	0.02	0.02	0.07	0.20	0.22	3.14	0	0	0
0.00	0.03	200	0.05	0.05	0.44	0.02	0.02	0.04	0.12	0.14	2.07	0	0	0
log(1.5)	0.20	50	0.08	0.09	0.45	0.02	0.02	0.07	0.16	0.17	2.11	0	0	0
log(1.5)	0.20	200	0.05	0.06	0.44	0.02	0.02	0.04	0.15	0.17	2.17	0	0	0
log(1.5)	0.03	50	0.08	0.09	0.51	0.02	0.02	0.07	0.15	0.17	2.99	0	0	0
log(1.5)	0.03	200	0.05	0.06	0.47	0.02	0.02	0.03	0.14	0.16	3.02	0	0	0

Table 7: The mean squared error (MSE) of the log(mOR) estimated using FLIC. Each row represents 495 scenarios with varying associations between the covariates and the exposure and/or outcome.

Marginal Risk Difference, Firth's corrected Logistic regression with intercept-correction

Values

			Mean			Min			Max			Warnings		
bYA	event fraction	nevents	Full	Selected	Unadj.	Full	Selected	Unadj.	Full	Selected	Unadj.	Full	Selected	Unadj.
0.0000	0.20	50	0.0169	0.0215	0.0803	-0.0035	-0.0010	0.0064	0.0594	0.0674	0.2467	0	0	0
0.0000	0.20	200	0.0188	0.0217	0.0753	-0.0042	-0.0015	0.0056	0.0628	0.0668	0.2098	0	0	0
0.0000	0.03	50	0.0032	0.0048	0.0416	-0.0029	-0.0008	0.0033	0.0111	0.0124	0.1713	0	0	0
0.0000	0.03	200	0.0035	0.0052	0.0490	-0.0021	-0.0003	0.0063	0.0117	0.0121	0.2080	0	0	0
log(1.5)	0.20	50	0.0215	0.0251	0.0885	-0.0033	0.0002	0.0145	0.0620	0.0655	0.2134	0	0	0
log(1.5)	0.20	200	0.0213	0.0241	0.0844	-0.0023	-0.0003	0.0171	0.0614	0.0660	0.2463	0	0	0
log(1.5)	0.03	50	0.0211	0.0241	0.0564	-0.0014	-0.0003	0.0058	0.0608	0.0672	0.2041	0	0	0
log(1.5)	0.03	200	0.0204	0.0232	0.0591	-0.0004	-0.0000	0.0032	0.0584	0.0652	0.2089	0	0	0

Table 8: The values of the mRD estimated using FLIC. Each row represents 495 scenarios with varying associations between the covariates and the exposure and/or outcome.

Bias

			Mean			Min			Max			Warnings		
bYA	event fraction	nevents	Full	Selected	Unadj.	Full	Selected	Unadj.	Full	Selected	Unadj.	Full	Selected	Unadj.
0.0000	0.20	50	-0.0016	0.0030	0.0619	-0.0099	-0.0017	0.0064	0.0035	0.0145	0.2004	0	0	0
0.0000	0.20	200	-0.0006	0.0023	0.0559	-0.0042	-0.0015	0.0056	0.0052	0.0111	0.1661	0	0	0
0.0000	0.03	50	-0.0003	0.0012	0.0380	-0.0029	-0.0008	0.0033	0.0018	0.0062	0.1713	0	0	0
0.0000	0.03	200	-0.0002	0.0016	0.0454	-0.0021	-0.0003	0.0033	0.0020	0.0078	0.2080	0	0	0
log(1.5)	0.20	50	-0.0006	0.0030	0.0663	-0.0033	-0.0006	0.0031	0.0037	0.0155	0.2027	0	0	0
log(1.5)	0.20	200	-0.0007	0.0021	0.0624	-0.0036	-0.0006	0.0058	0.0020	0.0070	0.2005	0	0	0
log(1.5)	0.03	50	-0.0019	0.0012	0.0334	-0.0090	-0.0040	0.0058	0.0006	0.0108	0.1539	0	0	0
log(1.5)	0.03	200	-0.0016	0.0012	0.0370	-0.0089	-0.0026	0.0032	0.0002	0.0087	0.1594	0	0	0

Table 9: The bias of the mRD estimated using FLIC. Each row represents 495 scenarios with varying associations between the covariates and the exposure and/or outcome.

Mean Squared Error

				Mean			Min			Max			Warnings	
bYA	event fraction	nevents	Full	Selected	Unadj.	Full	Selected	Unadj.	Full	Selected	Unadj.	Full	Selected	Unadj.
0.0000	0.20	50	0.0017	0.0019	0.0073	0.0000	0.0000	0.0001	0.0034	0.0036	0.0425	0	0	0
0.0000	0.20	200	0.0010	0.0011	0.0053	0.0000	0.0000	0.0000	0.0031	0.0034	0.0299	0	0	0
0.0000	0.03	50	0.0003	0.0003	0.0029	0.0001	0.0001	0.0001	0.0009	0.0009	0.03	0	0	0
0.0000	0.03	200	0.0003	0.0003	0.0047	0.0000	0.0000	0.0000	0.0009	0.0010	0.0438	0	0	0
log(1.5)	0.20	50	0.0011	0.0012	0.0077	0.0001	0.0001	0.0001	0.0036	0.0038	0.0435	0	0	0
log(1.5)	0.20	200	0.0005	0.0005	0.0063	0.0001	0.0001	0.0001	0.0009	0.0009	0.0408	0	0	0
log(1.5)	0.03	50	0.0009	0.0009	0.0027	0.0000	0.0000	0.0001	0.0034	0.0036	0.0260	0	0	0
log(1.5)	0.03	200	0.0008	0.0009	0.0033	0.0000	0.0000	0.0000	0.0031	0.0033	0.0278	0	0	0

Table 10: The mean squared error (MSE) of the mRD estimated using FLIC. Each row represents 495 scenarios with varying associations between the covariates and the exposure and/or outcome.

Conditional Odds Ratio, Firth's corrected Logistic regression with intercept-correction

Values

				Mean			Min			Max			Warnings	
bYA	event fraction	nevents	Full	Selected	Unadj.	Full	Selected	Unadj.	Full	Selected	Unadj.	Full	Selected	Unadj.
0.00	0.20	50	0.14	0.18	0.69	-0.03	-0.01	0.12	0.44	0.58	1.77	0	0	0
0.00	0.20	200	0.13	0.16	0.70	-0.03	-0.01	0.20	0.42	0.45	1.70	0	0	0
0.00	0.03	50	0.13	0.17	0.72	-0.02	-0.01	0.12	0.43	0.51	2.07	0	0	0
0.00	0.03	200	0.14	0.16	0.71	-0.03	-0.01	0.20	0.42	0.45	1.64	0	0	0
log(1.5)	0.20	50	0.27	0.31	0.81	-0.03	-0.00	0.19	0.44	0.48	1.61	0	0	0
log(1.5)	0.20	200	0.27	0.30	0.82	-0.02	-0.00	0.11	0.44	0.50	1.76	0	0	0
log(1.5)	0.03	50	0.27	0.31	0.85	-0.03	-0.00	0.20	0.44	0.53	1.69	0	0	0
log(1.5)	0.03	200	0.27	0.30	0.84	-0.01	0.00	0.11	0.44	0.51	2.08	0	0	0

Table 11: The values of the log(cOR) estimated using FLIC. Each row represents 495 scenarios with varying associations between the covariates and the exposure and/or outcome.

Bias

				Mean			Min			Max			Warnings	
bYA	event fraction	nevents	Full	Selected	Unadj.	Full	Selected	Unadj.	Full	Selected	Unadj.	Full	Selected	Unadj. 0.00
0.20	50	0.00	0.04	0.55	-0.03	-0.01	0.12	0.03	0.18	1.36	0	0	0	
0.00	0.20	200	-0.00	0.03	0.56	-0.03	-0.01	0.12	0.04	0.09	1.70	0	0	0
0.00	0.03	50	-0.00	0.03	0.58	-0.04	-0.01	0.12	0.03	0.10	1.66	0	0	0
0.00	0.03	200	0.00	0.03	0.58	-0.03	-0.01	0.12	0.02	0.09	1.43	0	0	0
log(1.5)	0.20	50	-0.00	0.04	0.54	-0.04	-0.01	0.12	0.03	0.13	1.40	0	0	0
log(1.5)	0.20	200	-0.00	0.03	0.55	-0.03	-0.00	0.11	0.03	0.09	1.35	0	0	0
log(1.5)	0.03	50	0.00	0.04	0.58	-0.03	-0.00	0.10	0.03	0.12	1.69	0	0	0
log(1.5)	0.03	200	0.00	0.03	0.57	-0.03	0.00	0.11	0.03	0.11	1.68	0	0	0

Table 12: The bias of the log(cOR) estimated using FLIC. Each row represents 495 scenarios with varying associations between the covariates and the exposure and/or outcome.

Mean Squared Error

				Mean			Min			Max			Warnings	
bYA	event fraction	nevents	Full	Selected	Unadj.	Full	Selected	Unadj.	Full	Selected	Unadj.	Full	Selected	Unadj.
0.00	0.20	50	0.12	0.13	0.44	0.02	0.02	0.07	0.24	0.31	2.01	0	0	0
0.00	0.20	200	0.07	0.08	0.44	0.02	0.02	0.04	0.22	0.25	2.92	0	0	0
0.00	0.03	50	0.09	0.10	0.48	0.03	0.03	0.07	0.26	0.28	2.98	0	0	0
0.00	0.03	200	0.06	0.06	0.44	0.02	0.02	0.04	0.15	0.17	2.07	0	0	0
log(1.5)	0.20	50	0.11	0.11	0.43	0.03	0.03	0.06	0.25	0.29	2.11	0	0	0
log(1.5)	0.20	200	0.06	0.07	0.41	0.03	0.03	0.04	0.18	0.20	1.87	0	0	0
log(1.5)	0.03	50	0.10	0.11	0.49	0.02	0.02	0.07	0.21	0.23	2.99	0	0	0
log(1.5)	0.03	200	0.07	0.08	0.44	0.02	0.02	0.03	0.23	0.27	2.86	0	0	0

Table 13: The mean squared error of the log(cOR) estimated using FLIC. Each row represents 495 scenarios with varying associations between the covariates and the exposure and/or outcome.

Coverage

				Coverage	
bYA	event fraction	nevents	Full	Selected	Unadj.
0.00	0.20	50	0.96	0.94	0.51
0.00	0.20	200	0.96	0.94	0.34
0.00	0.03	50	0.96	0.95	0.41
0.00	0.03	200	0.95	0.95	0.23
log(1.5)	0.20	50	0.96	0.94	0.49
log(1.5)	0.20	200	0.96	0.95	0.31
log(1.5)	0.03	50	0.96	0.95	0.39
log(1.5)	0.03	200	0.96	0.94	0.26

Table 14: The coverage of the log(cOR) estimated using FLIC based on the selected-model standard error of the exposure coefficient. Each row represents 495 scenarios with varying associations between the covariates and the exposure and/or outcome.

Maximum likelihood

Proportion of true confounders eliminated

			Proportion of true confounders eliminated		
bYA	event fraction	nevents	Full	Selected	Unadj.
0.00	0.20	50	0.00	0.73	1.00
0.00	0.20	200	0.00	0.72	1.00
0.00	0.03	50	0.00	0.74	1.00
0.00	0.03	200	0.00	0.72	1.00
log(1.5)	0.20	50	0.00	0.74	1.00
log(1.5)	0.20	200	0.00	0.73	1.00
log(1.5)	0.03	50	0.00	0.73	1.00
log(1.5)	0.03	200	0.00	0.72	1.00

Table 15: The proportion of true confounders eliminated by backward elimination in ML estimation.
Each row represents 495 scenarios with varying associations between the covariates and the exposure and/or outcome.

Marginal Risk Ratio estimated using Maximum Likelihood

Values

			Mean			Min			Max			Warnings		
bYA	event fraction	nevents	Full	Selected	Unadj.	Full	Selected	Unadj.	Full	Selected	Unadj.	Full	Selected	Unadj.
0.00	0.20	50	0.10	0.13	0.60	-0.02	-0.01	0.09	0.35	0.38	1.50	0	0	0
0.00	0.20	200	0.10	0.12	0.61	-0.02	-0.01	0.16	0.33	0.34	1.67	0	0	0
0.00	0.03	50	0.14	0.17	0.68	-0.02	-0.00	0.11	0.44	0.50	2.10	9	9	9
0.00	0.03	200	0.13	0.15	0.66	-0.02	-0.00	0.16	0.41	0.44	1.61	0	0	0
log(1.5)	0.20	50	0.23	0.26	0.72	-0.02	0.00	0.15	0.44	0.47	1.62	0	0	0
log(1.5)	0.20	200	0.23	0.25	0.73	-0.01	-0.00	0.09	0.44	0.48	1.70	0	0	0
log(1.5)	0.03	50	0.24	0.26	0.79	-0.02	0.01	0.20	0.41	0.44	1.70	0	0	0
log(1.5)	0.03	200	0.23	0.25	0.77	-0.01	0.00	0.11	0.40	0.45	2.05	1	1	1

Table 16: The values of the log(mRR) estimated using ML. Each row represents 495 scenarios with varying associations between the covariates and the exposure and/or outcome. In 10 datasets, the following warning occured when the unadjusted/full/selected model was fitted: "simpleWarning: glm.fit: fitted probabilities numerically 0 or 1 occurred". We discarded these datasets, which implies that 10 mRR estimates are missing.

Bias

			Mean			Min			Max			Warnings		
bYA	event fraction	nevents	Full	Selected	Unadj.	Full	Selected	Unadj.	Full	Selected	Unadj.	Full	Selected	Unadj.
0.00	0.20	50	0.01	0.03	0.50	-0.02	-0.01	0.09	0.04	0.11	1.28	0	0	0
0.00	0.20	200	0.00	0.02	0.51	-0.02	-0.01	0.10	0.04	0.07	1.67	0	0	0
0.00	0.03	50	0.01	0.04	0.56	-0.02	-0.00	0.11	0.06	0.14	1.75	9	9	9
0.00	0.03	200	0.01	0.03	0.53	-0.02	-0.00	0.12	0.03	0.10	1.31	0	0	0
log(1.5)	0.20	50	0.01	0.03	0.49	-0.02	0.00	0.13	0.05	0.09	1.25	0	0	0
log(1.5)	0.20	200	0.01	0.03	0.50	-0.01	-0.00	0.09	0.05	0.12	1.34	0	0	0
log(1.5)	0.03	50	0.01	0.04	0.57	-0.02	-0.00	0.09	0.04	0.12	1.70	0	0	0
log(1.5)	0.03	200	0.01	0.03	0.55	-0.01	0.00	0.11	0.04	0.07	1.70	1	1	1

Table 17: The bias of the log(mRR) estimated using ML. Each row represents 495 scenarios with varying associations between the covariates and the exposure and/or outcome. In 10 datasets, the following warning occured when the unadjusted/full/selected model was fitted: "simpleWarning: glm.fit: fitted probabilities numerically 0 or 1 occurred". We discarded these datasets, which implies that 10 mRR estimates are missing.

Mean Squared Error

				Mean			Min			Max			Warnings	
bYA	event fraction	nevents	Full	Selected	Unadj.	Full	Selected	Unadj.	Full	Selected	Unadj.	Full	Selected	Unadj.
0.00	0.20	50	0.07	0.07	0.36	0.02	0.02	0.07	0.13	0.13	1.71	0	0	0
0.00	0.20	200	0.04	0.04	0.37	0.01	0.01	0.03	0.11	0.10	2.82	0	0	0
0.00	0.03	50	0.08	0.09	0.46	0.01	0.01	0.04	0.23	0.25	3.30	9	9	9
0.00	0.03	200	0.05	0.05	0.37	0.01	0.01	0.03	0.13	0.14	1.85	0	0	0
log(1.5)	0.20	50	0.07	0.07	0.35	0.01	0.01	0.05	0.17	0.19	1.74	0	0	0
log(1.5)	0.20	200	0.05	0.05	0.35	0.01	0.01	0.02	0.17	0.19	1.96	0	0	0
log(1.5)	0.03	50	0.08	0.08	0.47	0.02	0.02	0.07	0.17	0.19	3.06	0	0	0
log(1.5)	0.03	200	0.05	0.05	0.42	0.02	0.02	0.03	0.12	0.12	2.96	1	1	1

Table 18: The MSE of the log(mRR) estimated using ML. Each row represents 495 scenarios with varying associations between the covariates and the exposure and/or outcome. In 10 datasets, the following warning occured when the unadjusted/full/selected model was fitted: "simpleWarning: glm.fit: fitted probabilities numerically 0 or 1 occurred". We discarded these datasets, which implies that 10 mRR estimates are missing.

Marginal Odds Ratio estimated using Maximum Likelihood

Values

			Mean			Min			Max			Warnings		
bYA	event fraction	nevents	Full	Selected	Unadj.	Full	Selected	Unadj.	Full	Selected	Unadj.	Full	Selected	Unadj.
0.00	0.20	50	0.13	0.15	0.70	-0.02	-0.01	0.12	0.43	0.46	1.82	0	0	0
0.00	0.20	200	0.13	0.15	0.70	-0.03	-0.01	0.20	0.41	0.43	1.71	0	0	0
0.00	0.03	50	0.14	0.17	0.73	-0.02	-0.01	0.12	0.45	0.51	2.15	9	9	9
0.00	0.03	200	0.14	0.16	0.72	-0.02	-0.00	0.21	0.42	0.45	1.65	0	0	0
log(1.5)	0.20	50	0.26	0.29	0.83	-0.02	0.00	0.19	0.45	0.49	1.66	0	0	0
log(1.5)	0.20	200	0.26	0.28	0.83	-0.01	-0.00	0.11	0.45	0.49	1.77	0	0	0
log(1.5)	0.03	50	0.26	0.29	0.86	-0.02	0.01	0.20	0.44	0.46	1.74	0	0	0
log(1.5)	0.03	200	0.26	0.28	0.85	-0.01	0.00	0.11	0.43	0.46	2.10	1	1	1

Table 19: The values of the log(mOR) estimated using ML. Each row represents 495 scenarios with varying associations between the covariates and the exposure and/or outcome. In 10 datasets, the following warning occured when the unadjusted/full/selected model was fitted: "simpleWarning: glm.fit: fitted probabilities numerically 0 or 1 occurred". We discarded these datasets, which implies that 10 mOR estimates are missing.

Bias

			Mean			Min			Max			Warnings		
bYA	event fraction	nevents	Full	Selected	Unadj.	Full	Selected	Unadj.	Full	Selected	Unadj.	Full	Selected	Unadj.
0.00	0.20	50	0.01	0.03	0.58	-0.02	-0.01	0.12	0.05	0.13	1.52	0	0	0
0.00	0.20	200	0.00	0.03	0.58	-0.03	-0.01	0.13	0.05	0.08	1.71	0	0	0
0.00	0.03	50	0.01	0.04	0.60	-0.02	-0.01	0.12	0.06	0.14	1.79	9	9	9
0.00	0.03	200	0.01	0.03	0.59	-0.02	-0.00	0.12	0.03	0.10	1.44	0	0	0
log(1.5)	0.20	50	0.01	0.04	0.58	-0.02	0.00	0.13	0.05	0.11	1.44	0	0	0
log(1.5)	0.20	200	0.01	0.03	0.58	-0.01	-0.00	0.11	0.05	0.12	1.47	0	0	0
log(1.5)	0.03	50	0.01	0.04	0.61	-0.02	-0.01	0.12	0.05	0.12	1.74	0	0	0
log(1.5)	0.03	200	0.01	0.03	0.60	-0.01	0.00	0.11	0.04	0.09	1.74	1	1	1

Table 20: The bias of the log(mOR) estimated using ML. Each row represents 495 scenarios with varying associations between the covariates and the exposure and/or outcome. In 10 datasets, the following warning occured when the unadjusted/full/selected model was fitted: "simpleWarning: glm.fit: fitted probabilities numerically 0 or 1 occurred". We discarded these datasets, which implies that 10 mOR estimates are missing.

Mean Squared Error

				Mean			Min			Max			Warnings	
bYA	event fraction	nevents	Full	Selected	Unadj.	Full	Selected	Unadj.	Full	Selected	Unadj.	Full	Selected	Unadj.
0.00	0.20	50	0.10	0.10	0.49	0.02	0.02	0.07	0.19	0.19	2.47	0	0	0
0.00	0.20	200	0.06	0.06	0.46	0.02	0.02	0.04	0.16	0.15	2.97	0	0	0
0.00	0.03	50	0.09	0.09	0.52	0.02	0.02	0.07	0.24	0.26	3.44	9	9	9
0.00	0.03	200	0.05	0.05	0.45	0.02	0.02	0.04	0.14	0.15	2.10	0	0	0
log(1.5)	0.20	50	0.09	0.09	0.47	0.02	0.02	0.07	0.18	0.20	2.22	0	0	0
log(1.5)	0.20	200	0.06	0.06	0.46	0.02	0.02	0.04	0.18	0.20	2.21	0	0	0
log(1.5)	0.03	50	0.10	0.09	0.53	0.02	0.02	0.07	0.19	0.20	3.21	0	0	0
log(1.5)	0.03	200	0.06	0.06	0.49	0.02	0.02	0.03	0.17	0.17	3.09	1	1	1

Table 21: The MSE of the log(mOR) estimated using ML. Each row represents 495 scenarios with varying associations between the covariates and the exposure and/or outcome. In 10 datasets, the following warning occured when the unadjusted/full/selected model was fitted: “simpleWarning: glm.fit: fitted probabilities numerically 0 or 1 occurred”. We discarded these datasets, which implies that 10 mOR estimates are missing.

Marginal Risk Difference estimated using Maximum Likelihood

Values

				Mean			Min			Max			Warnings	
bYA	event fraction	nevents	Full	Selected	Unadj.	Full	Selected	Unadj.	Full	Selected	Unadj.	Full	Selected	Unadj.
0.0000	0.20	50	0.0186	0.0219	0.0817	-0.0040	-0.0013	0.0064	0.0661	0.0690	0.2511	0	0	0
0.0000	0.20	200	0.0193	0.0218	0.0760	-0.0047	-0.0018	0.0056	0.0645	0.0673	0.2107	0	0	0
0.0000	0.03	50	0.0034	0.0050	0.0420	-0.0030	-0.0009	0.0033	0.0117	0.0129	0.1720	9	9	9
0.0000	0.03	200	0.0036	0.0053	0.0493	-0.0022	-0.0002	0.0064	0.0119	0.0122	0.2089	0	0	0
log(1.5)	0.20	50	0.0221	0.0253	0.0895	-0.0038	-0.0001	0.0148	0.0637	0.0661	0.2143	0	0	0
log(1.5)	0.20	200	0.0219	0.0244	0.0849	-0.0025	-0.0003	0.0174	0.0632	0.0666	0.2474	0	0	0
log(1.5)	0.03	50	0.0230	0.0248	0.0573	-0.0011	-0.0001	0.0059	0.0678	0.0693	0.2078	0	0	0
log(1.5)	0.03	200	0.0222	0.0238	0.0599	-0.0004	0.0000	0.0032	0.0650	0.0671	0.2126	1	1	1

Table 22: The values of the log(mRD) estimated using ML. Each row represents 495 scenarios with varying associations between the covariates and the exposure and/or outcome. In 10 datasets, the following warning occured when the unadjusted/full/selected model was fitted: “simpleWarning: glm.fit: fitted probabilities numerically 0 or 1 occurred”. We discarded these datasets, which implies that 10 mRD estimates are missing.

Bias

				Mean			Min			Max			Warnings	
bYA	event fraction	nevents	Full	Selected	Unadj.	Full	Selected	Unadj.	Full	Selected	Unadj.	Full	Selected	Unadj.
0.0000	0.20	50	0.0001	0.0034	0.0632	-0.0041	-0.0013	0.0064	0.0042	0.0151	0.2048	0	0	0
0.0000	0.20	200	-0.0001	0.0024	0.0566	-0.0047	-0.0018	0.0056	0.0057	0.0106	0.1691	0	0	0
0.0000	0.03	50	-0.0001	0.0015	0.0384	-0.0030	-0.0009	0.0033	0.0018	0.0062	0.1720	9	9	9
0.0000	0.03	200	-0.0001	0.0017	0.0457	-0.0022	-0.0002	0.0034	0.0020	0.0077	0.2089	0	0	0
log(1.5)	0.20	50	-0.0000	0.0031	0.0673	-0.0038	-0.0002	0.0034	0.0039	0.0147	0.2063	0	0	0
log(1.5)	0.20	200	-0.0001	0.0024	0.0629	-0.0025	-0.0003	0.0061	0.0020	0.0075	0.2016	0	0	0
log(1.5)	0.03	50	0.0001	0.0018	0.0343	-0.0043	-0.0026	0.0059	0.0059	0.0121	0.1576	0	0	0
log(1.5)	0.03	200	0.0001	0.0017	0.0379	-0.0032	-0.0009	0.0032	0.0045	0.0099	0.1631	1	1	1

Table 23: The bias of the log(mRD) estimated using ML. Each row represents 495 scenarios with varying associations between the covariates and the exposure and/or outcome. In 10 datasets, the following warning occured when the unadjusted/full/selected model was fitted: “simpleWarning: glm.fit: fitted probabilities numerically 0 or 1 occurred”. We discarded these datasets, which implies that 10 mRD estimates are missing.

Mean Squared Error

				Mean			Min			Max			Warnings	
bYA	event fraction	nevents	Full	Selected	Unadj.	Full	Selected	Unadj.	Full	Selected	Unadj.	Full	Selected	Unadj.
0.0000	0.20	50	0.0021	0.0020	0.0077	0.0000	0.0000	0.0001	0.0041	0.0039	0.0443	0	0	0
0.0000	0.20	200	0.0012	0.0012	0.0054	0.0000	0.0000	0.0000	0.0039	0.0036	0.0310	0	0	0
0.0000	0.03	50	0.0003	0.0003	0.0030	0.0001	0.0001	0.0001	0.0009	0.0009	0.0302	9	9	9
0.0000	0.03	200	0.0003	0.0003	0.0047	0.0000	0.0000	0.0000	0.0010	0.0010	0.0442	0	0	0
log(1.5)	0.20	50	0.0013	0.0013	0.0079	0.0001	0.0001	0.0001	0.0045	0.0041	0.0451	0	0	0
log(1.5)	0.20	200	0.0005	0.0005	0.0064	0.0001	0.0001	0.0001	0.0009	0.0009	0.0412	0	0	0
log(1.5)	0.03	50	0.0011	0.0010	0.0028	0.0000	0.0000	0.0001	0.0041	0.0039	0.0272	0	0	0
log(1.5)	0.03	200	0.0010	0.0010	0.0035	0.0000	0.0000	0.0000	0.0038	0.0035	0.0291	1	1	1

Table 24: The MSE of the log(mRD) estimated using ML. Each row represents 495 scenarios with varying associations between the covariates and the exposure and/or outcome. In 10 datasets, the following warning occured when the unadjusted/full/selected model was fitted: "simpleWarning: glm.fit: fitted probabilities numerically 0 or 1 occurred". We discarded these datasets, which implies that 10 mRD estimates are missing.

Conditional Odds Ratio estimated using Maximum Likelihood

Values

				Mean			Min			Max			Warnings	
bYA	event fraction	nevents	Full	Selected	Unadj.	Full	Selected	Unadj.	Full	Selected	Unadj.	Full	Selected	Unadj.
0.00	0.20	50	0.16	0.18	0.70	-0.03	-0.01	0.12	0.53	0.62	1.82	0	0	0
0.00	0.20	200	0.14	0.16	0.70	-0.04	-0.02	0.20	0.43	0.46	1.71	0	0	0
0.00	0.03	50	0.15	0.18	0.73	-0.02	-0.01	0.12	0.58	0.68	2.27	9	9	9
0.00	0.03	200	0.14	0.17	0.72	-0.03	-0.00	0.21	0.43	0.46	1.65	0	0	0
log(1.5)	0.20	50	0.28	0.31	0.83	-0.03	-0.00	0.19	0.47	0.51	1.66	0	0	0
log(1.5)	0.20	200	0.29	0.31	0.83	-0.02	-0.00	0.11	0.48	0.54	1.77	0	0	0
log(1.5)	0.03	50	0.30	0.32	0.86	-0.03	0.01	0.20	0.51	0.56	1.74	0	0	0
log(1.5)	0.03	200	0.30	0.31	0.85	-0.01	0.00	0.11	0.53	0.55	2.10	1	1	1

Table 25: The values of the log(cOR) estimated using ML. Each row represents 495 scenarios with varying associations between the covariates and the exposure and/or outcome. In 10 datasets, the following warning occured when the unadjusted/full/selected model was fitted: "simpleWarning: glm.fit: fitted probabilities numerically 0 or 1 occurred". We discarded these datasets, which implies that 10 mRR estimates are missing.

Bias

				Mean			Min			Max			Warnings	
bYA	event fraction	nevents	Full	Selected	Unadj.	Full	Selected	Unadj.	Full	Selected	Unadj.	Full	Selected	Unadj.
0.00	0.20	50	0.03	0.05	0.57	-0.03	-0.01	0.12	0.13	0.21	1.41	0	0	0
0.00	0.20	200	0.00	0.03	0.57	-0.04	-0.02	0.12	0.05	0.09	1.71	0	0	0
0.00	0.03	50	0.01	0.04	0.60	-0.02	-0.01	0.12	0.17	0.27	1.87	9	9	9
0.00	0.03	200	0.00	0.03	0.58	-0.03	-0.00	0.12	0.03	0.11	1.44	0	0	0
log(1.5)	0.20	50	0.01	0.04	0.56	-0.03	-0.00	0.13	0.06	0.13	1.44	0	0	0
log(1.5)	0.20	200	0.02	0.04	0.56	-0.02	-0.00	0.11	0.07	0.13	1.36	0	0	0
log(1.5)	0.03	50	0.03	0.05	0.59	-0.03	0.00	0.11	0.10	0.15	1.74	0	0	0
log(1.5)	0.03	200	0.03	0.04	0.58	-0.01	0.00	0.11	0.12	0.14	1.70	1	1	1

Table 26: The bias of the log(cOR) estimated using ML. Each row represents 495 scenarios with varying associations between the covariates and the exposure and/or outcome. In 10 datasets, the following warning occured when the unadjusted/full/selected model was fitted: "simpleWarning: glm.fit: fitted probabilities numerically 0 or 1 occurred". We discarded these datasets, which implies that 10 mRR estimates are missing.

Mean Squared Error

				Mean			Min			Max			Warnings	
bYA	event fraction	nevents	Full	Selected	Unadj.	Full	Selected	Unadj.	Full	Selected	Unadj.	Full	Selected	Unadj.
0.00	0.20	50	0.16	0.14	0.46	0.02	0.02	0.07	0.40	0.38	2.16	0	0	0
0.00	0.20	200	0.10	0.09	0.45	0.02	0.02	0.04	0.33	0.29	2.97	0	0	0
0.00	0.03	50	0.12	0.12	0.53	0.03	0.03	0.07	4.02	4.08	7.51	9	9	9
0.00	0.03	200	0.06	0.06	0.45	0.02	0.02	0.04	0.18	0.19	2.10	0	0	0
log(1.5)	0.20	50	0.14	0.13	0.45	0.03	0.03	0.06	0.39	0.34	2.22	0	0	0
log(1.5)	0.20	200	0.07	0.07	0.42	0.03	0.03	0.04	0.22	0.23	1.96	0	0	0
log(1.5)	0.03	50	0.13	0.12	0.51	0.02	0.02	0.07	0.32	0.28	3.21	0	0	0
log(1.5)	0.03	200	0.10	0.09	0.46	0.02	0.02	0.03	1.37	1.33	2.93	1	1	1

Table 27: The MSE of the log(cOR) estimated using ML. Each row represents 495 scenarios with varying associations between the covariates and the exposure and/or outcome. In 10 datasets, the following warning occured when the unadjusted/full/selected model was fitted: "simpleWarning: glm.fit: fitted probabilities numerically 0 or 1 occurred". We discarded these datasets, which implies that 10 mRR estimates are missing.

Coverage

				Coverage	
bYA	event fraction	nevents	Full	Selected	Unadj.
0.00	0.20	50	0.94	0.93	0.49
0.00	0.20	200	0.94	0.94	0.33
0.00	0.03	50	0.95	0.94	0.39
0.00	0.03	200	0.95	0.94	0.22
log(1.5)	0.20	50	0.94	0.94	0.47
log(1.5)	0.20	200	0.95	0.94	0.30
log(1.5)	0.03	50	0.95	0.94	0.37
log(1.5)	0.03	200	0.95	0.94	0.24

Table 28: The coverage of the log(cOR) estimated using Maximum Likelihood based on the selected-model standard error of the exposure coefficient. Each row represents 495 scenarios with varying associations between the covariates and the exposure and/or outcome.

Descriptives simulation data

Frequency of exposure A

bYA	event fraction	nevents	Mean (sd)	Min	Max
0.00	0.20	50	0.5(0.032)	0.35	0.64
0.00	0.20	200	0.5(0.016)	0.43	0.57
0.00	0.03	50	0.5(0.012)	0.45	0.56
0.00	0.03	200	0.5(0.006)	0.47	0.53
log(1.5)	0.20	50	0.5(0.032)	0.35	0.64
log(1.5)	0.20	200	0.5(0.016)	0.43	0.58
log(1.5)	0.03	50	0.5(0.012)	0.44	0.56
log(1.5)	0.03	200	0.5(0.006)	0.47	0.53

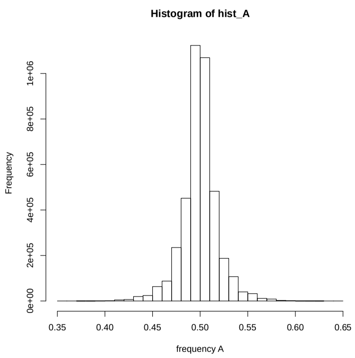

Frequency of outcome Y

bYA	event fraction	nevents	Mean (sd)	Min	Max
0.00	0.20	50	0.2(0.025)	0.09	0.32
0.00	0.20	200	0.2(0.013)	0.14	0.26
0.00	0.03	50	0.03(0.004)	0.01	0.05
0.00	0.03	200	0.03(0.002)	0.02	0.04
log(1.5)	0.20	50	0.2(0.025)	0.09	0.32
log(1.5)	0.20	200	0.2(0.013)	0.15	0.26
log(1.5)	0.03	50	0.03(0.004)	0.01	0.05
log(1.5)	0.03	200	0.03(0.002)	0.02	0.04

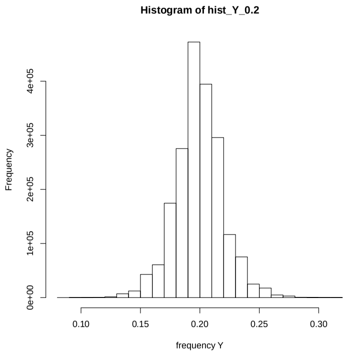

Supplement: Supplementary file 4 — Supporting information [file BIMJ-66-0-s001.pdf]
