# Supplementary material for: A comparison of full model specification and backward elimination of potential confounders when estimating marginal and conditional causal effects on binary outcomes from observational data
Source: Biom J. 2022 May 12;66(1):2100237. doi: 10.1002/bimj.202100237 (PMC10952199; doi:10.1002/bimj.202100237)
Supplement: Supplementary file 5 — Supporting information [file BIMJ-66-0-s002.zip › Code_Data/README_Replication_Explainer.html]

Replication\_Explainer


# Replication\_Explainer

The overall computational resources needed to replicate the entire simulation study are large. Therefore, this file describes how to replicate a single simulation scenario of choice using the code of the original simulation. The replicated results can be compared to the results from the original simulation, which are saved under ./data/original\_summarised.  
It takes around 45 minutes to run a single scenario from the first group (scenarios 1:495).

## Select a scenario

The first lines of code in the execute\_simstudy.R file can be used to recreate the matrix of all simulation scenarios (object called “all\_datagen\_scenarios”)

```
source( file = "./rcode/packages/packages.R",
        local = knitr::knit_global())
source( file = "./rcode/dgm/create_dirs.R",
        local = knitr::knit_global())
source( file = "./rcode/sim/run_sim.R",
        local = knitr::knit_global())
source( file = "./rcode/sumsim/summarize_simulation.R",
        local = knitr::knit_global())
source( file = "./rcode/sumsim/diagnostics.R",
        local = knitr::knit_global())
source( file = "./rcode/visualisation/simresults_table.R",
        local = knitr::knit_global())

# Select datagen_scenarios and analysis_scenarios to be used
all_datagen_scenarios <- datagen_scenarios()  # Redundant step, used for testing
```

Inspect the simulation scenarios and select a scenario of interest, for instance, the first scenario.

```
head( all_datagen_scenarios)
```

```
##   nevents nL bYA   bL_const eventrate rhoL       bAL1       bYL1 bAL2 bYL2 bAL3
## 1      50 24   0 0.04879016       0.2  0.3 0.00000000 0.00000000    0    0    0
## 2      50 24   0 0.04879016       0.2  0.3 0.00000000 0.04879016    0    0    0
## 3      50 24   0 0.04879016       0.2  0.3 0.00000000 0.18232156    0    0    0
## 4      50 24   0 0.04879016       0.2  0.3 0.04879016 0.00000000    0    0    0
## 5      50 24   0 0.04879016       0.2  0.3 0.04879016 0.04879016    0    0    0
## 6      50 24   0 0.04879016       0.2  0.3 0.04879016 0.18232156    0    0    0
##   bYL3 bAL4 bYL4      Yint scen_num
## 1    0    0    0 -1.423828        1
## 2    0    0    0 -1.443604        2
## 3    0    0    0 -1.521240        3
## 4    0    0    0 -1.420166        4
## 5    0    0    0 -1.447998        5
## 6    0    0    0 -1.515381        6
```

```
all_datagen_scenarios[1,]
```

```
##   nevents nL bYA   bL_const eventrate rhoL bAL1 bYL1 bAL2 bYL2 bAL3 bYL3 bAL4
## 1      50 24   0 0.04879016       0.2  0.3    0    0    0    0    0    0    0
##   bYL4      Yint scen_num
## 1    0 -1.423828        1
```

Now store the selected scenario to run the simulation for these parameter inputs. The number of repetitions in the original simulation was 1000, but this can be altered, too.

```
# The simulation scenario selected for replication
# Warning: scenarios 2476 - 2970 and 3467 - 3960 can take more than a day to finish!!
# the fastest scenarios to simulate are 1:495 (50 events, eventrate 0.20) - they take about 1 hour
# select a scenario here:
selected_scenario <- 1

# Set other parameters needed for execute_simstudy.R file
use_datagen_scenarios <- all_datagen_scenarios[selected_scenario, ]
use_analysis_scenarios <- analysis_scenarios()
# Set number of simulations to perform (1000 was used in the paper):
rep <-1000
```

## Show stored simulation results

In the following code chunk the saved simulation results for the selected simulation scenario are obtained and shown, for comparison with the reproduced simulations:

```
# read saved results for all simulation scenarios
# results for FLIC
results_FLIC_A <- readRDS(paste0("./data/original_summarised/FLIC_0.157_A.rds"))
results_FLIC_mRR <- readRDS(paste0("./data/original_summarised/FLIC_0.157_MRR.rds"))
results_FLIC_mOR <- readRDS(paste0("./data/original_summarised/FLIC_0.157_MOR.rds"))
# results for ML
results_ML_A <- readRDS(paste0("./data/original_summarised/ML_0.157_A.rds"))
results_ML_mRR <- readRDS(paste0("./data/original_summarised/ML_0.157_MRR.rds"))
results_ML_mOR <- readRDS(paste0("./data/original_summarised/ML_0.157_MOR.rds"))

# read the results for the selected simulation scenario
results_FLIC_A[ results_FLIC_A$scen_num == selected_scenario,]
```

```
##    scen_num          model     method      value_A       bias_A   empSE_A
## 1:        1           full FLIC_0.157 -0.006021297 -0.006021297 0.3396805
## 2:        1 selected_0.157 FLIC_0.157  0.007043933  0.007043933 0.3539641
## 3:        1     unadjusted FLIC_0.157  0.115000686  0.115000686 0.3159471
##      empvar_A     MSE_A removed_replications warnings_count warnings_message
## 1: 0.11538285 0.1153037                    0              0               NA
## 2: 0.12529057 0.1252149                    0              0               NA
## 3: 0.09982256 0.1129479                    0              0               NA
```

```
results_FLIC_mRR[ results_FLIC_mRR$scen_num == selected_scenario,]
```

```
##    scen_num          model     method    value_MRR     bias_MRR empSE_MRR
## 1:        1           full FLIC_0.157 -0.002998838 -0.002998838 0.2449643
## 2:        1 selected_0.157 FLIC_0.157  0.006700647  0.006700647 0.2619021
## 3:        1     unadjusted FLIC_0.157  0.092186675  0.092186675 0.2546427
##    empvar_MRR    MSE_MRR removed_replications warnings_count warnings_message
## 1: 0.06000752 0.05995650                    0              0               NA
## 2: 0.06859270 0.06856901                    0              0               NA
## 3: 0.06484292 0.07327646                    0              0               NA
```

```
results_FLIC_mOR[ results_FLIC_mRR$scen_num == selected_scenario,]
```

```
##    scen_num          model     method    value_MOR     bias_MOR empSE_MOR
## 1:        1           full FLIC_0.157 -0.003987178 -0.003987178 0.3044221
## 2:        1 selected_0.157 FLIC_0.157  0.008172358  0.008172358 0.3250127
## 3:        1     unadjusted FLIC_0.157  0.115000686  0.115000686 0.3159471
##    empvar_MOR    MSE_MOR removed_replications warnings_count warnings_message
## 1: 0.09267279 0.09259602                    0              0               NA
## 2: 0.10563326 0.10559441                    0              0               NA
## 3: 0.09982256 0.11294790                    0              0               NA
```

```
results_ML_A[ results_ML_A$scen_num == selected_scenario,]
```

```
##    scen_num          model   method      value_A       bias_A   empSE_A
## 1:        1           full ML_0.157 -0.007330849 -0.007330849 0.3942780
## 2:        1 selected_0.157 ML_0.157  0.004853329  0.004853329 0.3705131
## 3:        1     unadjusted ML_0.157  0.117044998  0.117044998 0.3217855
##     empvar_A     MSE_A removed_replications warnings_count warnings_message
## 1: 0.1554551 0.1553534                    0              0               NA
## 2: 0.1372800 0.1371662                    0              0               NA
## 3: 0.1035459 0.1171419                    0              0               NA
```

```
results_ML_mRR[ results_ML_mRR$scen_num == selected_scenario,]
```

```
##    scen_num          model   method    value_MRR     bias_MRR empSE_MRR
## 1:        1           full ML_0.157 -0.003181999 -0.003181999 0.2754047
## 2:        1 selected_0.157 ML_0.157  0.005333708  0.005333708 0.2713789
## 3:        1     unadjusted ML_0.157  0.093844853  0.093844853 0.2594529
##    empvar_MRR    MSE_MRR removed_replications warnings_count warnings_message
## 1: 0.07584773 0.07578201                    0              0               NA
## 2: 0.07364650 0.07360130                    0              0               NA
## 3: 0.06731581 0.07605535                    0              0               NA
```

```
results_ML_mOR[ results_ML_mRR$scen_num == selected_scenario,]
```

```
##    scen_num          model   method    value_MOR     bias_MOR empSE_MOR
## 1:        1           full ML_0.157 -0.004313284 -0.004313284 0.3418252
## 2:        1 selected_0.157 ML_0.157  0.006442845  0.006442845 0.3366732
## 3:        1     unadjusted ML_0.157  0.117044998  0.117044998 0.3217855
##    empvar_MOR   MSE_MOR removed_replications warnings_count warnings_message
## 1:  0.1168445 0.1167463                    0              0               NA
## 2:  0.1133488 0.1132770                    0              0               NA
## 3:  0.1035459 0.1171419                    0              0               NA
```

## Rerun simulation scenario

The following code chunk reruns the simulation for the selected scenario:

```
# This code is copied from ./rcode/execute_simstudy.R

# Create filestructure  ----
#------------------------------------------------------------------------------#

dirpaths  <- lapply(create_dirpaths(use_analysis_scenarios = analysis_scenarios()),
       function(x) dir.create(x,recursive = TRUE))
```

```
## Warning in dir.create(x, recursive = TRUE): '.\data\raw\truth' existiert bereits
```

```
filepaths <- lapply(create_dirpaths(use_analysis_scenarios = analysis_scenarios()),
                    FUN = function(x) create_filepaths(dirpaths = x,
                                                       use_datagen_scenarios = use_datagen_scenarios))

invisible(lapply(unlist(filepaths),
                 FUN= function(x) saveRDS(NULL,file=x)))


# Run simulation study  ----
#------------------------------------------------------------------------------#
# run_sim()
run_sim(rep = rep,
        use_datagen_scenarios = use_datagen_scenarios,
        use_analysis_scenarios = use_analysis_scenarios)

# Summarize processed simulation output  ----
#------------------------------------------------------------------------------#
# summarize_sim()
invisible(apply(use_analysis_scenarios,
                MARGIN = 1,
                FUN = function(x) sum_multiple_scenarios(
                  method = x[['method']],
                  pcutoff = x[['pcutoff']],
                  use_datagen_scenarios = use_datagen_scenarios,
                  estimator = "A",
                  rep = rep)))
```

```
## Warning in dir.create(file.path(".", "data", "summarised"), recursive = TRUE):
## '.\data\summarised' existiert bereits
```

```
## Warning in dir.create(file.path(".", "data", "summarised"), recursive = TRUE):
## '.\data\summarised' existiert bereits
```

```
invisible(apply(use_analysis_scenarios,
                MARGIN = 1,
                FUN = function(x) sum_multiple_scenarios(
                  method = x[['method']],
                  pcutoff = x[['pcutoff']],
                  use_datagen_scenarios = use_datagen_scenarios,
                  estimator = "MOR",
                  rep = rep)))
```

```
## Warning in dir.create(file.path(".", "data", "summarised"), recursive = TRUE):
## '.\data\summarised' existiert bereits

## Warning in dir.create(file.path(".", "data", "summarised"), recursive = TRUE):
## '.\data\summarised' existiert bereits
```

```
invisible(apply(use_analysis_scenarios,
                MARGIN = 1,
                FUN = function(x) sum_multiple_scenarios(
                  method = x[['method']],
                  pcutoff = x[['pcutoff']],
                  use_datagen_scenarios = use_datagen_scenarios,
                  estimator = "MRR",
                  rep = rep)))
```

```
## Warning in dir.create(file.path(".", "data", "summarised"), recursive = TRUE):
## '.\data\summarised' existiert bereits

## Warning in dir.create(file.path(".", "data", "summarised"), recursive = TRUE):
## '.\data\summarised' existiert bereits
```

## Present the replicated results in a similar form to the results from the original simulation

For comparison with the stored results, here the reproduced simulation results are presented:

```
# results for FLIC
replicated_results_FLIC_A <- readRDS(paste0("./data/summarised/FLIC_0.157_A.rds"))
replicated_results_FLIC_mRR <- readRDS(paste0("./data/summarised/FLIC_0.157_MRR.rds"))
replicated_results_FLIC_mOR <- readRDS(paste0("./data/summarised/FLIC_0.157_MOR.rds"))
# results for ML
replicated_results_ML_A <- readRDS(paste0("./data/summarised/ML_0.157_A.rds"))
replicated_results_ML_mRR <- readRDS(paste0("./data/summarised/ML_0.157_MRR.rds"))
replicated_results_ML_mOR <- readRDS(paste0("./data/summarised/ML_0.157_MOR.rds"))

# read the results for the selected simulation scenario
replicated_results_FLIC_A[ replicated_results_FLIC_A$scen_num == selected_scenario,]
```

```
##    scen_num          model     method      value_A       bias_A   empSE_A
## 1:        1           full FLIC_0.157 -0.006021289 -0.006021289 0.3396804
## 2:        1 selected_0.157 FLIC_0.157  0.007043944  0.007043944 0.3539640
## 3:        1     unadjusted FLIC_0.157  0.115000687  0.115000687 0.3159471
##      empvar_A     MSE_A removed_replications warnings_count warnings_message
## 1: 0.11538277 0.1153036                    0              0               NA
## 2: 0.12529055 0.1252149                    0              0               NA
## 3: 0.09982256 0.1129479                    0              0               NA
```

```
replicated_results_FLIC_mRR[ replicated_results_FLIC_mRR$scen_num == selected_scenario,]
```

```
##    scen_num          model     method    value_MRR     bias_MRR empSE_MRR
## 1:        1           full FLIC_0.157 -0.002998833 -0.002998833 0.2449642
## 2:        1 selected_0.157 FLIC_0.157  0.006700655  0.006700655 0.2619021
## 3:        1     unadjusted FLIC_0.157  0.092186675  0.092186675 0.2546427
##    empvar_MRR    MSE_MRR removed_replications warnings_count warnings_message
## 1: 0.06000748 0.05995647                    0              0               NA
## 2: 0.06859270 0.06856900                    0              0               NA
## 3: 0.06484292 0.07327646                    0              0               NA
```

```
replicated_results_FLIC_mOR[ replicated_results_FLIC_mRR$scen_num == selected_scenario,]
```

```
##    scen_num          model     method    value_MOR     bias_MOR empSE_MOR
## 1:        1           full FLIC_0.157 -0.003987172 -0.003987172 0.3044220
## 2:        1 selected_0.157 FLIC_0.157  0.008172368  0.008172368 0.3250127
## 3:        1     unadjusted FLIC_0.157  0.115000687  0.115000687 0.3159471
##    empvar_MOR    MSE_MOR removed_replications warnings_count warnings_message
## 1: 0.09267274 0.09259597                    0              0               NA
## 2: 0.10563324 0.10559440                    0              0               NA
## 3: 0.09982256 0.11294790                    0              0               NA
```

```
replicated_results_ML_A[ replicated_results_ML_A$scen_num == selected_scenario,]
```

```
##    scen_num          model   method      value_A       bias_A   empSE_A
## 1:        1           full ML_0.157 -0.007330849 -0.007330849 0.3942780
## 2:        1 selected_0.157 ML_0.157  0.004853329  0.004853329 0.3705131
## 3:        1     unadjusted ML_0.157  0.117045000  0.117045000 0.3217855
##     empvar_A     MSE_A removed_replications warnings_count warnings_message
## 1: 0.1554551 0.1553534                    0              0               NA
## 2: 0.1372800 0.1371662                    0              0               NA
## 3: 0.1035459 0.1171419                    0              0               NA
```

```
replicated_results_ML_mRR[ replicated_results_ML_mRR$scen_num == selected_scenario,]
```

```
##    scen_num          model   method    value_MRR     bias_MRR empSE_MRR
## 1:        1           full ML_0.157 -0.003181999 -0.003181999 0.2754047
## 2:        1 selected_0.157 ML_0.157  0.005333708  0.005333708 0.2713789
## 3:        1     unadjusted ML_0.157  0.093844854  0.093844854 0.2594529
##    empvar_MRR    MSE_MRR removed_replications warnings_count warnings_message
## 1: 0.07584773 0.07578201                    0              0               NA
## 2: 0.07364650 0.07360130                    0              0               NA
## 3: 0.06731581 0.07605535                    0              0               NA
```

```
replicated_results_ML_mOR[ replicated_results_ML_mRR$scen_num == selected_scenario,]
```

```
##    scen_num          model   method    value_MOR     bias_MOR empSE_MOR
## 1:        1           full ML_0.157 -0.004313284 -0.004313284 0.3418252
## 2:        1 selected_0.157 ML_0.157  0.006442845  0.006442845 0.3366732
## 3:        1     unadjusted ML_0.157  0.117045000  0.117045000 0.3217855
##    empvar_MOR   MSE_MOR removed_replications warnings_count warnings_message
## 1:  0.1168445 0.1167463                    0              0               NA
## 2:  0.1133488 0.1132770                    0              0               NA
## 3:  0.1035459 0.1171419                    0              0               NA
```
